# Childhood diarrhoea attributed to enteropathogenic bacteria in low- and middle-income countries: a systematic review and meta-analysis

**DOI:** 10.7189/jogh.15.04350

**Published:** 2025-11-28

**Authors:** Md Abu Sayeed, Samantha Colquhoun, Stefan Thottunkal, Angus McLure, Alice Richardson, Aparna Lal, Md Rezanur Rahaman

**Affiliations:** 1 National Centre for Epidemiology and Population Health, The Australian National University, Canberra, Australian Capital Territory, Australia; 2 Statistical Support Network, The Australian National University, Canberra, Australian Capital Territory, Australia

## Abstract

**Background:**

Diarrhoea among children under five years old (*i.e.* childhood diarrhoea) causes significant morbidity and mortality in low and middle-income countries (LMICs). We conducted this systematic review and meta-analysis to quantify the proportion of childhood diarrhoea attributable to *Campylobacter *spp., diarrhoeagenic* Escherichia coli (E. coli)*, *Salmonella* spp., and *Shigella* spp. in LMIC settings.

**Methods:**

We included epidemiological studies published between 2000 and 2025, and extracted data on study location, sample size, and pathogen-specific parameters. Two reviewers independently performed database searches, publication screening, data extraction, and quality assessment, with any conflicts resolved by a third reviewer. We reported the results of the meta-analysis as pooled proportions (positive samples divided by the total samples tested for each enteropathogen) and 95% confidence intervals. We assessed all potential sources of heterogeneity using univariable and multivariable meta-regression, quantified moderator contributions as pseudo-R^2^ values based on reductions in *τ*^2^, and tested for interaction effects. We also evaluated robustness through leave-one-out analyses, sequentially excluding individual studies to examine their influence on pooled estimates and heterogeneity.

**Results:**

We included 71 records encompassing 84 studies. Pooled proportions were 8.6% for *Campylobacter* spp., 23.0% for diarrhoeagenic *E. coli* (DEC), 2.6% for *Salmonella* spp., and 8.8% for *Shigella* spp., with wide variation across studies. Subgroup analyses showed higher proportions of *Campylobacter* spp. and *Shigella* spp. in Asia and with polymerase chain reaction-based detection, and greater DEC proportions in America and Africa. *Salmonella* spp. remained low across regions and study designs. Year- and country-specific analyses showed no consistent temporal trends, though DEC peaked in 2014 (77.9%, two studies) and *Shigella* spp. was higher in 2020 (20.7%, ten studies), both driven by a limited number of studies.

**Conclusions:**

Standardising diagnostic methods and study designs is essential for reducing heterogeneity and improving the reliability of pooled proportion estimates in epidemiological research on enteric pathogens. Improving water supply, sanitation, hygiene, and food safety remains crucial for reducing the burden of childhood diarrhoea in LMICs.

Diarrhoea in children under five years of age (*i.e.* childhood diarrhoea) remains a serious global health challenge, claiming the lives of about 9% of young children worldwide, with the heaviest burden falling on those in low- and middle-income countries (LMICs) [1]. Poor sanitation, unsafe drinking water, inadequate hygiene, and limited access to health care make children in these settings especially vulnerable [2]. Childhood diarrhoea can stem from a range of infectious agents (*e.g.* bacteria, viruses, and parasites) and non-infectious causes. Among these, certain bacteria, including *Campylobacter* spp., diarrhoeagenic *Escherichia coli* (DEC), *Salmonella* spp., and *Shigella* spp., are some of the most common culprits [3–6].

These bacterial infections often present with some common symptoms like watery or bloody diarrhoea, abdominal pain, fever, nausea, and vomiting, though each pathogen has its own distinctive features. While *Campylobacter jejuni* and *Campylobacter coli* frequently cause inflammatory diarrhoea with fever and severe abdominal cramps [7], *Salmonella* spp. like *Salmonella enterica* can lead to enteroinvasive infections characterised by high fever, abdominal pain, and diarrhoea [8]. The various DEC strains that include enteropathogenic, enterotoxigenic, enteroaggregative, enteroinvasive, shiga toxin-producing or enterohaemorrhagic, and diffusely adherent *E. coli* are major causes of acute watery diarrhoea, and some can lead to haemorrhagic colitis depending on their virulence [9]. *Shigella*, a leading cause of bacillary dysentery, is highly contagious, especially in settings with poor sanitation, and can cause severe illness in malnourished or immunocompromised children [10]. While many of these infections are mild and self-limiting, they can be deadly in very young, malnourished, or immunosuppressed children [11,12]. Understanding both their shared and unique clinical presentations is crucial for timely diagnosis and effective treatment.

Over the years, several studies have explored the burden of childhood diarrhoea and its risk factors, highlighting the significant role of these bacteria [13–15]. However, these studies differ from one another in scope, methods, and regional focus, and none have comprehensively quantified the prevalence of *Campylobacter*, DEC, *Salmonella*, and *Shigella* specifically in children from LMICs. We aim to fill this gap by performing a systematic review and meta-analysis that would provide robust, up-to-date pooled prevalence estimates for these key pathogens. Our findings could offer a valuable evidence base to guide targeted interventions and policies aimed at reducing the toll of childhood diarrhoea.

The zoonotic nature of these bacteria and the importance of a One Health perspective are well documented [16]. Building on this understanding, this review systematically quantifies the burden of these infections in children under five years of age, providing foundational evidence to guide future research on transmission dynamics, risk factors, and One Health-oriented interventions. *Campylobacter* infections are often linked to the consumption of contaminated food, especially undercooked poultry, and contact with infected animals, such as domestic poultry [17,18]. Similarly, *Salmonella*, transmitted from poultry, cattle, and pigs to humans, underscores the necessity to consider both animal and human health in diarrhoeal disease management [19]. Shigellosis, an occupational primate-associated zoonosis, is also transmitted *via* the faecal-oral route through contaminated water (*e.g.* animal faeces) as it can spread even through small amounts of contaminated faeces, and through person-to-person transmission occurring in regions with overcrowding, poor sanitation, and inadequate personal hygiene [20–22]. Children under five, particularly those in childcare settings and schools with poor sanitation, are at high risk of shigellosis, along with travellers to areas with unsafe water and food, and displaced people [23]. DEC is transmitted by both contaminated food and water [24].

Addressing childhood diarrhoea in LMICs requires a clear understanding of how much each of these pathogens contributes to the overall burden. Previous studies have shown that these bacteria can spread through multiple environmental pathways, including contaminated water, soil, surfaces, and close contact with animals [25,26]. While this review does not directly assess risk factors or transmission routes, its findings on pathogen-specific proportion provide a foundation for future studies and interventions within a One Health framework. The complex interaction of social, environmental, and behavioural factors calls for coordinated efforts across human health, animal health, and environmental sectors. Our review takes an important step in this direction by synthesising evidence on the proportion of *Campylobacter* spp., DEC, *Salmonella* spp., and *Shigella* spp., building on earlier reviews and addressing critical gaps in our understanding.

## METHODS

We registered our review in PROSPERO (CRD42023473909) and reported its findings according to the PRISMA guidelines [27].

### Eligibility criteria

We considered epidemiological studies focussing on childhood diarrhoea in LMICs, as classified by the World Bank in 2023 (low income, lower middle income, middle income, and poor countries) [28]. Childhood diarrhoea was defined as the passing of three or more loose or liquid stools per day, with acute diarrhoea lasting less than seven days, persistent diarrhoea lasting 14 days or longer, and chronic diarrhoea lasting 28 days or longer [11,29].

We considered original research articles with the following inclusion criteria (Table S1 in the **Online Supplementary Document**):

– cross-sectional, cohort, case-control, and experimental studies available in full text and published in English between January 2000 and January 2025;

– study population comprised of children with diarrhoea under five years old and residing in LMICs [28];

– childhood diarrhoea cases tested for one or more of the following enteropathogenic bacteria: *Campylobacter* spp., DEC, *Salmonella* spp., or *Shigella* spp.

We included studies where diarrhoeal stool samples were collected from children under five years of age and excluded those that used rectal swabs or unspecified sample types, as well as those that did not report both the number of diarrhoeal stool samples tested and the number of positive detections for each pathogen. We also excluded systematic reviews and meta-analyses, but did screen their reference lists to identify additional eligible primary studies. We also did not consider grey literature, conference abstracts, dissertations, and non-peer-reviewed materials to ensure methodological rigor and reliability of the findings.

### Search strategy

We developed a logic grid using indexing languages (Emtree, MeSH) search terms, and/or keywords for each database and search engine (PubMed, Web of Science, Embase OVID, Scopus, ProQuest, and Google Scholar), in consultation with an experienced information specialist to ensure comprehensiveness and reproducibility (Table S2 in the **Online Supplementary Document**). Major keywords included children, diarrhoea, enteropathogens, *i.e.* DEC, *Campylobacter* spp., *Salmonella* spp., *Shigella* spp.; study designs such as observational, intervention, case-control, cross-sectional, and cohort studies; and low- and middle-income countries.

We first ran an initial search in April 2024, followed by an updated one in January 2025 to capture new publications. All databases were accessed through the Australian National University (ANU) Library subscriptions. Backward citation searches were later conducted using the Systematic Review Accelerator [30], following citation chains up to two degrees of separation. This process leveraged the Lens repository, which indexes a vast literature base, covering PubMed, PubMed Central, CrossRef, Microsoft Academic Graph, and CORE databases.

### Selection of publication and data extraction

#### Publication selection

We downloaded all search results into EndNote, version 21 (Clarivate, London, UK) and exported them into Covidence (Veritas Health Innovation, Melbourne, Australia) for screening. Following deduplication, two reviewers (MAS, ST) performed the initial title and abstract screening, after which they reviewed the full texts of the eligible records. The second reviewer (ST) performed forward and backwards searching. The titles and abstracts, and subsequently the full texts of the records identified through forward and backward searches were also screened independently by both reviewers (MAS, ST). Finally, the first reviewer (MAS) extracted the data from the final selected publications, with cross-checks conducted by the other reviewers.

During database searching, the reviewers achieved an observed agreement of 92% at the title and abstract screening stage and 94.5% at the full-text screening stage, with Cohen’s kappa (κ) values of 0.84 and 0.89 indicating strong and almost perfect agreement, respectively. Among the records identified through forward and backward citation searching, the observed agreement was 91.2% at the title and abstract screening stage (κ ≈ 0.82) and 95% at the full-text screening stage (κ ≈ 0.90), also indicating strong to almost perfect agreement. Disagreements were resolved through discussion and consensus in all stages (Table S3 in the **Online Supplementary Document**).

#### Quality assessment of individual publications

We conducted the quality assessment of each included publication using Joanna Briggs Institute (JBI) tools [31]. Assessment criteria included appropriateness of the sampling frame, sampling methods, and adequacy of sample size. It also covered a detailed description of study subjects and study settings, the number of samples that were identified in the study and used in data analysis, the use of valid identification methods for the standard and reliable measurement of the outcome, appropriate statistical analysis, and management of low response rates if applicable (Table S4 in the **Online Supplementary Document**). To ensure consistency, two reviewers independently piloted the JBI checklist on a subset of studies, compared scoring decisions, and refined the interpretation of criteria before proceeding to full quality assessment. Discrepancies during the main assessment were resolved through discussion and consensus.

#### Data extraction and recording

We developed and piloted a data extraction Excel spreadsheet on five randomly selected studies to ensure clarity, completeness, and consistency across reviewers (Table S5 in the **Online Supplementary Document**). Based on this piloting, we added a column for diagnostic methods before full data extraction commenced. When a research article presented multiple study findings from different locations (*i.e.* when each location had its own unique study), we extracted the data separately. The final data extraction sheet captured details under multiple headings from each study, including study location, citation details, first author, title, study period, year of publication, sample size, number of positive samples for each organism, and author conclusion. We also extracted reported diagnostic methods (*e.g.* culture and PCR) used in each study. Where studies reported DEC results at the pathotype level (*e.g.* enteroaggregative, enteropathogenic, enteroinvasive, and shiga-toxin producing enterohaemorrhagic), we extracted these data separately to enable pathotype-specific analyses. The primary outcome was the proportion of diarrhoeal stool samples positive for each pathogen, expressed as a percentage of total diarrhoeal stool samples tested. This ensured consistency across studies, regardless of reporting format. All included studies specified their diagnostic methods, and therefore, no assumptions or imputations were required. Although included studies differed in design (cross-sectional, case-control, and cohort), we harmonised extraction to ensure comparability. Specifically, for case-control studies, we extracted data related to diarrhoeal cases in children under five years of age and their corresponding stool samples, while excluding controls without diarrhoea. For cohort studies, we considered only stool samples collected at the first diarrhoeal episode from children under five years and excluded data related to follow-up samples. For cross-sectional study, we included the cases only. This approach meant that across all designs, extracted data were conceptually equivalent to cross-sectional samples of diarrhoeal cases tested for pathogens. This standardisation minimised heterogeneity introduced by study design differences and created a more homogeneous data set for pooling.

### Statistical analysis

#### Descriptive and meta-analysis

We first performed a descriptive analysis and reported the study characteristics using frequency, number, and percentage in a tabular form. Then, we conducted the enteropathogen-specific meta-analysis using *R*, version 4.0.2 (R Core Team, Vienna, Austria). To ensure stability in the variance for meta-analyses of single proportions, we transformed proportions using the logit-transformation method [32] and fitted generalised linear mixed models with a random-effects structure and Hartung-Knapp adjustment, implemented using the *R* ‘meta’ package, version 2.4-0 [33–35]. After estimation, we back-transformed final pooled proportions and their corresponding 95% confidence intervals (CIs) to their original values and expressed them as percentages for ease of interpretation and comparability across studies. Pooled proportion estimates were calculated and represented in percentage values by continent, country, and year of publication. We also performed a separate meta-analysis for DEC pathotypes. Between-study heterogeneity was quantified using *τ*^2^ (between-study variance), the *I*^2^ statistic (percentage of total variability due to heterogeneity), and Cochran’s Q test. We applied a random-effects model throughout, as we anticipated heterogeneity *a priori*, given the diversity of settings, populations, and diagnostic methods. A schematic flow diagram of the analysis is provided in Figure S1 in the **Online Supplementary Document**.

#### Sources of heterogeneity and sub-group analysis

We explored sources of heterogeneity by conducting subgroup analyses and meta-regression across seven study-level characteristics, including study design, population source, continent, diagnostic methods, study duration (percentile), sample size (percentile), and study year. We independently examined all factors in univariable analysis to determine their contribution to between-study heterogeneity, forwarding variables with *P* ≤ 0.05 into the multiple meta-regression model [36] and reported coefficients and *P* values in a tabular form. To quantify moderator contributions, we calculated pseudo-R^2^ based on the reduction in *τ*^2^ compared with the baseline model [37]. Additionally, we assessed interaction effects between variables in the multivariable meta-regression model. Sensitivity analysis was then performed by using the leave-one-out approach, sequentially excluding each study to examine its influence on pooled estimates.

#### Publication bias

We evaluated publication bias and small study effects using three complementary approaches: Egger’s regression test, Begg’s rank correlation test, and Doi plots with the Luis Furuya Kanamori (LFK) index [38,39]. For Egger’s and rank correlation tests, statistical significance was set at *P* ≤ 0.05. The LFK index was interpreted as within ±1 indicating no asymmetry, between ±1 and ±2 indicating minor asymmetry, and greater than ±2 indicating major asymmetry.

## RESULTS

### Publication selection

**Figure 1:**
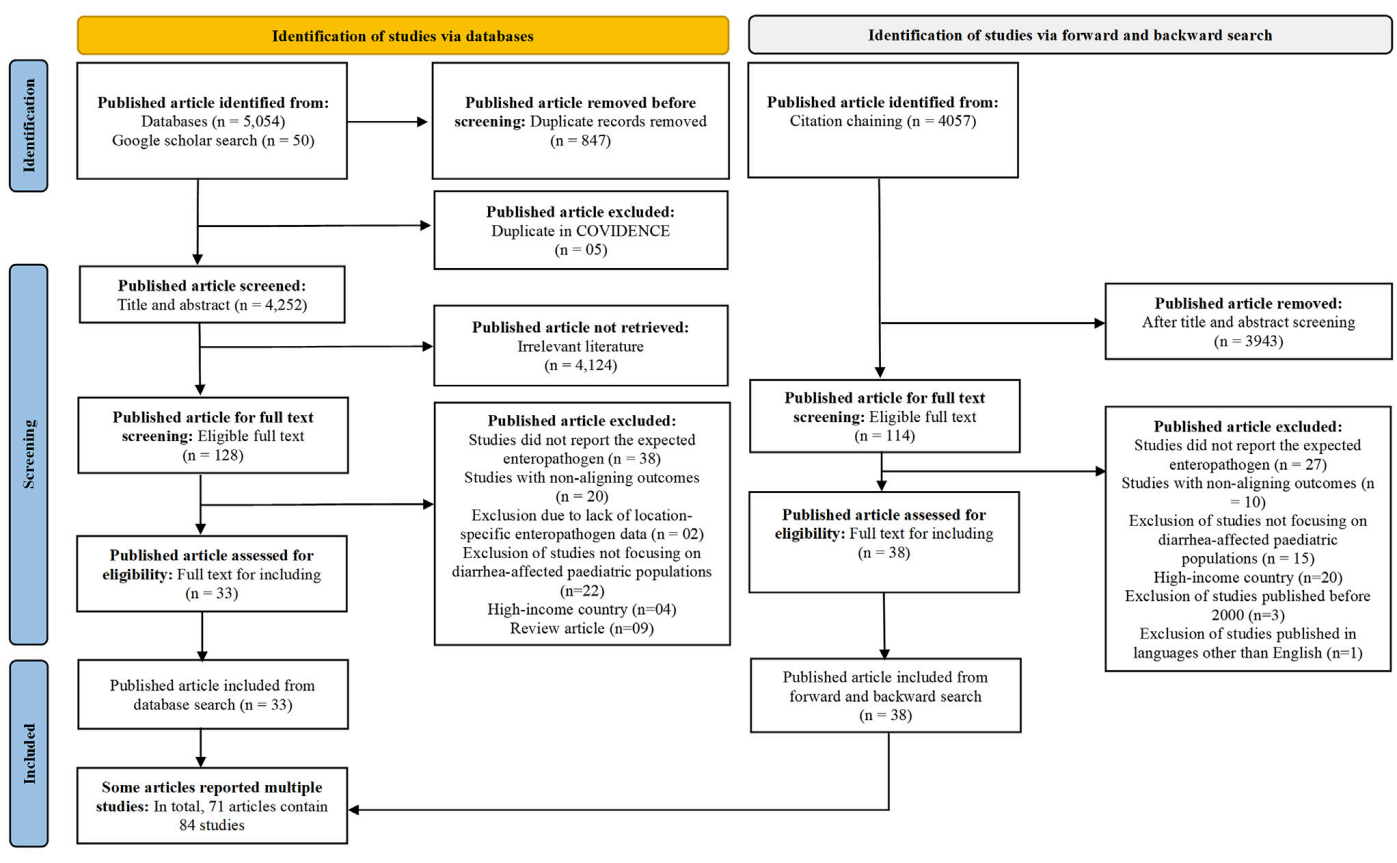
PRISMA diagram showing publication selection process for this review.

We identified 5104 records in total: 5054 from the initial database search and an additional 50 from the Google Scholar search. Screening of titles and abstracts yielded 128 eligible for full-text review (**Figure 1**), of which 33 (25.8%) were selected for data extraction. This subset of 33 records was entered into the SRA, which conducted an unfiltered forward and backward search and identified 4057 additional records. These, too, underwent title and abstract screening, with 114 retained for full-text review, of which 38 (33.3%) were selected for data extraction. We finally included 71 publications presenting 84 studies, as some reported on multiple studies (**Figure 1**; **Online Supplementary Document**). We observed that 53.5% of the included publications were identified through the forward and backward search due to the sheer extensiveness of the Lens repository. Among the included publications, 40 (56.3%) were classified as ‘very strong’, 29 (40.8%) as ‘strong’, and 2 (2.8%) as ‘average’ in quality, according to the JBI’s quality assessment criteria (Table S4 in the **Online Supplementary Document**).

### Study characteristics

Of the 84 included studies, 54 (64.3%) were published between 2016 and 2024, 24 (28.6%) from 2008 to 2015, and 6 (7.1%) from 2000 to 2007. Most studies (n = 59, 70.2%) were conducted in the African region, with the highest number coming from Ethiopia (n = 18, 21.4%). *Campylobacter* spp. was reported in 38 (45.2%), DEC in 46 (54.8%), *Salmonella* spp. in 53 (63.1%), and *Shigella* spp. in 60 (71.4%) studies (**Table 1**).

**Table 1:** Characteristics of studies included in the review

	Frequency (%)	References
**Year of study**		
2000–07	6 (7.1)	[40–45]
2008–15	24 (28.6)	[3,46–68]
2016–24	54 (64.3)	[69-109]
**Continent**		
Africa	59 (70.2)	[3,42,44,45,47,49–51,53–57,59–79,81–84,89,90,92,94–96,98–101,103–106,108,109]
Asia	21 (25.0)	[40,41,43,46,52,58,85–88,91–93,95,97,102]
America	4 (4.8)	[48,80,92,107]
**Sampling frame**		
0–12 mo	41 (48.8)	[3,41–46,49–52,54,55,58–60,62–66,68,71,73,77–79,81,84,86,87,94,97–101,106–109]
13–24 mo	9 (10.7)	[40,47,48,53,57,69,72,96,104]
≥25 mo	34 (40.5)	[56,61,67,70,74–76,80,82,83,85,87-93,95,102,103,105]
**Economic status**		
Lower middle income	43 (51.2)	[40–43,45–47,50,52,57,58,60,64,70–74,76,85-89,91–93,95,97,98,100,102,103,105,107,108]
Low income	38 (45.2)	[3,44,49,51,53-56,59,61–63,65–69,75,77–79,81–84,90,94–96,99,101,104–106,109]
Middle income	3 (3.6)	[48,80,92]
**Study design**		
Cross-sectional study	46 (54.8)	[3,40–42,44,45,47,49–52,55,57,59–66,68,70,71,73,74,76–79,81,84,86,88,91,93,94,97–101,104,106,108,109]
Case-control study	30 (35.7)	[43,46,53,54,56,58,67,69,72,75,80,82,83,85,89,90,95,96,102,103,105,107]
Cohort study	8 (9.5)	[48,87,92]
**Bacterial pathogens**		
*Campylobacter* spp	38 (45.2)	[3,41,42,44,47,48,53–57,60–62,64,67-69,71,73,74,80,82,83,85–88,96,98,99,101,102,104,105,109]
DEC	46 (54.8)	[40–46,48,50–54,57,61,64,66–71,73-76,78,81–83,85,87–90,93,96,97,102–105,107,108]
*Salmonella* spp	53 (63.1)	[3,42,44,47,50–54,57–59,61,63,64,66–70,72,73,76–79,81–85,87,88,91,93–97,100,102–106]
*Shigella* spp	60 (71.4)	[3,41–44,46–54,57,59,61,63–74,76–85,87,88,92–94,96–98,100,102,104–106,108]
**Organism diagnostic methods**		
PCR	27 (32.1)	[40,42,43,56,61,71,74,75,78,80,86–90,92,96,105,107,108]
Culture-based methods (*e.g.* culturing, isolation, staining, and biochemical test)	57 (67.9)	[3,41,44–55,57–60,62–70,72,73,76,77,79,81–85,91,93–95,97–104,106,109]
**Country**		
Bangladesh	6 (7.1)	[40,85,87,92,95,97]
Burkina Faso	2 (2.4)	[51,54]
Central African Republic	1 (1.2)	[69]
Ethiopia	18 (21.4)	[3,49,55,59,62,63,65,66,77,79,81,84,94,99,101,104,106,109]
Gambia	3 (3.6)	[75,95,105]
Ghana	1 (1.2)	[72]
Guinea-Bissau	1 (1.2)	[96]
Haiti	1 (1.2)	[107]
India	6 (7.1)	[86,88,91–93,95]
Kenya	8 (9.5)	[47,70,76,89,95,98,100,105]
Madagascar	1 (1.2)	[53]
Malawi	1 (1.2)	[56]
Mali	2 (2.4)	[95,105]
Morocco	1 (1.2)	[64]
Mozambique	6 (7.1)	[44,67,82,83,90,95]
Nepal	3 (3.6)	[52,92,102]
Nigeria	1 (1.2)	[103]
Pakistan	2 (2.4)	[92,95]
Peru	3 (3.6)	[48,80,92]
Rwanda	1 (1.2)	[61]
Senegal	1 (1.2)	[57]
Sudan	2 (2.4)	[68,78]
Tanzania	7 (8.3)	[42,45,50,60,71,74,92]
Vietnam	4 (4.8)	[41,43,46,58]
Zambia	2 (2.4)	[73,108]

PCR – polymerase chain reaction

### Proportion of *Campylobacter* spp. among childhood diarrhoeal patients

**Figure 2:**
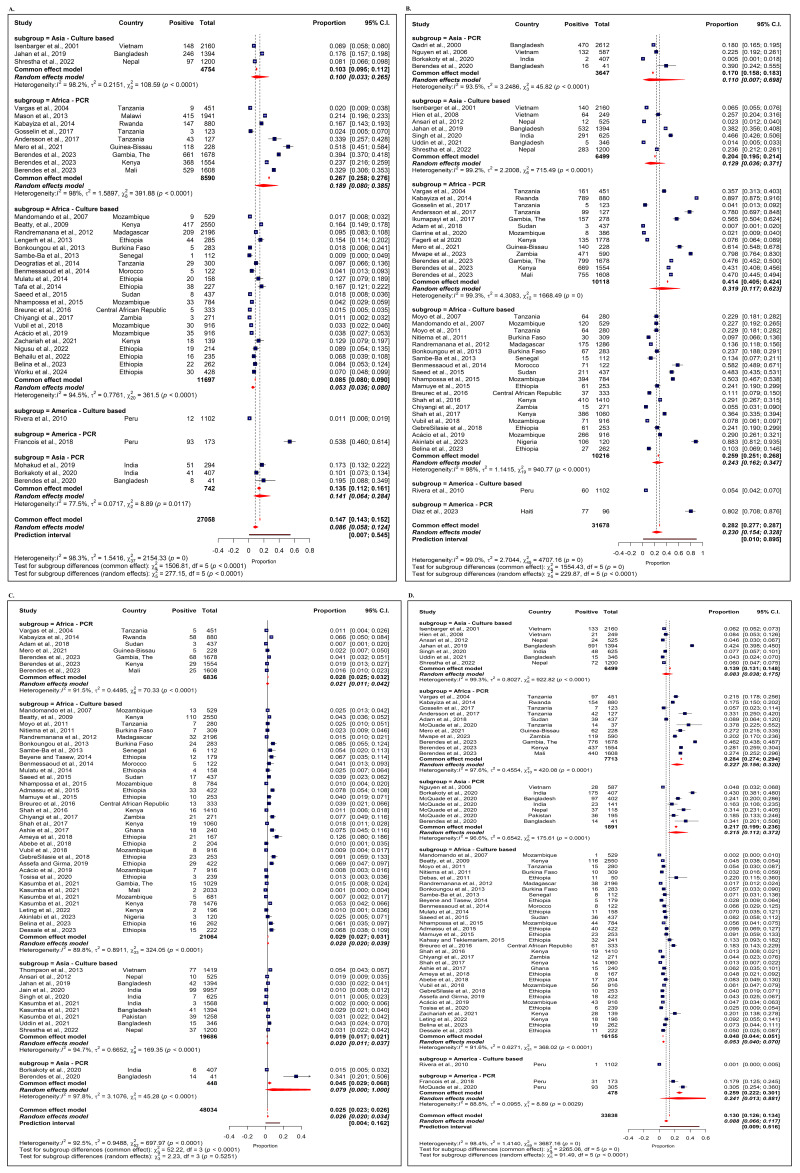
Forest plot illustrating the proportion of different enteric pathogens at the individual study level and detection method level by continent. The navy squares indicate the point estimate, and the red diamonds the contributions by individual study weight in the meta-analysis, the navy square with a long horizontal line indicates a wide 95% CI, and the navy square with a plus sign indicates a narrow 95% CI of the point estimate, while the red line indicates the PI. **Panel A. ***Campylobacter *spp. **Panel B. **DEC. **Panel C. ***Salmonella *spp. **Panel D. ***Shigella *spp.

Across 38 studies, the pooled proportion of *Campylobacter* spp. among diarrhoea-affected children’s stool sample was 8.6% (95% confidence interval (CI) = 5.8–12.4), with high heterogeneity (*I*^2^ = 98.3%) and a wide prediction interval (PI) (0.7–54.5%). Estimates differed markedly by diagnostic method. In Africa, the pooled meta-analysis estimates for polymerase chain reaction (PCR)-based studies were 18.9% (95% CI = 8.0–38.5%), compared with 5.3% (95% CI = 3.6–8.0) in culture-based studies. In Asia, pooled estimates were 14.1% (95% CI = 6.4–28.4) for PCR-based studies *vs*. 10.0% (95% CI = 3.3–26.5) for culture method-based studies. In the Americas, PCR-based estimates were substantially higher (53.8%; 95% CI = 46.0–61.4%) than in culture-based studies (1.1%; 95% CI = 0.6–1.9); however, they were based only on a single study in each category (**Figure 2**, Panel A).

### Proportion of DEC among childhood diarrhoeal patients

From 46 studies, the estimated pooled proportion of DEC among diarrhoeal stool samples of children under five years of age was 23.0% (95% CI = 15.4–32.8), with very high heterogeneity (*I*^2^ = 99.0%) and a broad PI (1.0–89.5%). The diagnostic method strongly influenced the results. In Africa, estimates for PCR-based detection showed a higher pooled proportion of DEC (31.9%; 95% CI = 11.7–62.3) compared with culture-based detection (24.3%; 95% CI = 16.2–34.7). In Asia, the pooled estimates for PCR-based studies yielded 11.0% (95% CI = 0.7–69.8), while those for the culture-based studies were 12.9% (95% CI = 3.6–37.1). In the Americas, a single PCR study reported estimates of 80.2% (95% CI = 70.8–87.6), compared with 5.4% (95% CI = 4.2–7.0) from a culture-based study (**Figure 2**, Panel B).

When analysed by pathotype, enteroaggregative *E. coli* showed the highest pooled proportion (15.8%; 95% CI = 9.7–24.8), followed by enterotoxigenic (8.1%; 95% CI = 5.0–12.9) and enteropathogenic (7.8%; 95% CI = 4.8–12.5) *E. coli*. Enteroinvasive and shiga-toxin producing or enterohaemorrhagic *E. coli* were rarely detected, with pooled proportions of 0.5% (95% CI = 0.2–1.2) and 0.7% (95% CI = 0.2–2.0), respectively. We noted considerable heterogeneity across all subgroups (*I*^2 ^≥ 90%) and wide prediction intervals, highlighting the variability in study estimates (Table S6 in the **Online Supplementary Document**).

### Proportion of *Salmonella *spp. among childhood diarrhoeal patients

Based on 53 studies, the pooled proportion of *Salmonella* spp. was 2.6% (95% CI = 2.0–3.4) among diarrhoeal stool samples, with substantial heterogeneity (*I*^2^ = 92.5) and a wide PI (0.4–16.2%). Proportions were generally low across both diagnostic methods, but pooled meta-analysis estimates for PCR-based detection showed a higher proportion of *Salmonella* spp. positive samples among diarrhoea-affected children. In Africa, the pooled estimate for PCR-based detection was 2.1% (95% CI = 1.1–4.2), compared with 2.8% (95% CI = 2.0–3.9) for culture-based detection. In Asia, pooled culture-based estimates were 2.0% (95% CI = 1.1–3.7), while those for PCR-based detection were 7.9% (95% CI = 0.0–100), although this estimate was based on only two studies (**Figure 2**, Panel C).

### Proportion of *Shigella *spp. among childhood diarrhoeal patients

Across 60 studies, the pooled proportion of *Shigella* spp. in diarrhoeal stool sample was 8.8% (95% CI = 6.6–11.7%), with very high heterogeneity (*I*^2^ = 98.4%) and a wide PI (0.9–51.6%). Diagnostic method was a key contributor to variability. In Africa, pooled meta-analysis estimates for PCR-based detection of *Shigella* spp. were 22.7% (95% CI = 15.6–32.0), compared with pooled culture-based estimates of 5.3% (95% CI = 4.0–7.0). In Asia, the pooled estimate for PCR-based detection was 21.5% (95% CI = 11.2–37.2), while pooled culture-based estimates were 8.3% (95% CI = 3.8–17.5). In the Americas, the pooled estimate for PCR-based detection was 24.1% (95% CI = 1.3–88.1), while those for single culture-based studies was 0.1% (95% CI = 0.0–0.5) (**Figure 2**, Panel D).

### Subgroup analysis and sources of heterogeneity

#### Subgroup analysis of enteric pathogen proportions across key study variables

The proportion of *Campylobacter* spp. was the highest in Asia (11.8%; 95% CI = 7.5–18.0%, PI = 5.0–25.4%, six studies) followed by America (10.1%; 95% CI = 0.0–100.0%, PI = 0.0–96.9%, two studies) and Africa (7.8%; 95% CI = 5.0–12.0%, PI = 0.7–49.9%, 30 studies). Case-control studies reported higher proportion of *Campylobacter* spp. (12.4%; 95% CI = 5.9–24.3%, PI = 0.8–70.9%, 14 studies) compared to cross-sectional (7.2%; 95% CI = 4.7–10.8%, PI = 1.1–35.3%, 22 studies) and cohort studies (4.6%; 95% CI = 0.0–100.0%, PI = 0.1–64.7%, two studies). PCR-based detection identified a higher pooled proportion of *Campylobacter* spp. (20.0%; 95% CI = 11.0–33.5%, PI = 2.4–72.0%, 13 studies) compared to culture-based methods (5.4%; 95% CI = 3.7–7.9%, PI = 0.9–26.5%, 25 studies). Community-based samples reported a higher pooled proportion of *Campylobacter* spp. (12.1%; 95% CI = 4.7–28.1%, PI = 0.7–72.5%, 10 studies) than hospital-based samples (7.6%; 95% CI = 5.0–11.4%, PI = 0.9–43.6%, 28 studies) (**Table 2**).

**Table 2:** Subgroup analysis of enteric pathogen proportions by different predictors*

	*Campylobacter* spp.	DEC	*Salmonella* spp.	*Shigella* spp.
**Continent**				
Africa	7.8 (5.0–12.0, 0.7–49.9, 30)	27.3 (17.8–39.5, 1.7–89.1, 33)	2.7 (2.0–3.6, 0.4–14.2, 41)	7.9 (5.7–10.7, 1.0–41.9, 43)
Asia	11.8 (7.5–18.0, 5.0–25.4, 6)	12.2 (4.5–29.2, 0.5–78.5, 11)	2.5 (1.2–5.2, 0.2–21.9, 12)	13.6 (8.0–22.2, 2.0–55.1, 14)
America	10.1 (0.0–100.0, 0.0–96.9, 2)	32.5 (0.0–100.0, 0.3–98.8, 2)		4.3 (0.0–98.1, 0.0–96.1, 3)
**Study design**				
Cross-sectional	7.2 (4.7–10.8, 1.1–35.3, 22)	18.3 (9.5–32.4, 0.6–89.5, 25)	3.0 (2.2–4.1, 0.7–12.8, 30)	6.8 (4.9–9.3, 1.0–33.7, 36)
Case–control	12.4 (5.9–24.3, 0.8–70.9, 14)	31.1 (19.0–46.4, 2.9–87.2, 19)	1.9 (1.2–3.0, 0.3–12.3, 22)	11.5 (6.7–19.0, 1.4–54.5, 16)
Cohort	4.6 (0.0–100.0, 0.1–64.7, 2)	15.5 (0.0–100.0, 1.1–75.9, 2)	34.1 (21.4–49.7, –, 1)	15.6 (3.8–46.4, 0.4–89.0, 8)
**Population source**				
Community	12.1 (4.7–28.1, 0.7–72.5, 10)	27.5 (13.0–49.0, 2.2–86.4, 11)	2.8 (0.7–10.7, 0.2–32.3, 6)	13.6 (6.5–26.4, 0.7–77.4, 16)
Hospital	7.6 (5.0–11.4, 0.9–43.6, 28)	21.7 (13.3–33.4, 0.9–89.4, 35)	2.6 (2.0–3.4, 0.4–14.1, 47)	7.5 (5.6–10.0, 1.1–36.7, 44)
**Detection method**				
Culture-based	5.4 (3.7–7.9, 0.9–26.5, 25)	19.9 (13.3–28.8, 2.1–74.8, 28)	2.6 (1.9–3.5, 0.4–14.4, 44)	5.3 (3.9–7.2, 0.8–27.8, 40)
PCR	20.0 (11.0–33.5, 2.4–72.0, 13)	28.2 (12.0–53.1, 0.6–96.5, 18)	2.7 (1.1–6.7, 0.2–24.1, 9)	22.4 (17.0–28.8, 6.6–54.2, 20)
**Study duration**				
P50 (≤median)	7.1 (4.5–11.2, 1.0–36.1, 19)	20.9 (11.6–34.6, 1.1–86.5, 23)	4.0 (2.8–5.6, 0.7–19.1, 27)	7.2 (5.3–9.9, 1.3–31.0, 31)
P100 (>median)	10.2 (5.4–18.5, 0.7–66.1, 19)	25.3 (14.0–41.3, 1.1–90.8, 23)	1.7 (1.2–2.5, 0.3–8.9, 26)	10.9 (6.7–17.3, 0.7–66.4, 29)

*All values are presented as pooled proportion (confidence interval, prediction interval, number of studies) unless specified otherwise. Prediction intervals were not estimable for single-study countries (one study); for very sparse strata (two or fewer studies), they should be interpreted with caution due to instability.

The pooled proportion for DEC was highest in America (32.5%; 95% CI = 0.0–100.0%, PI = 0.3–98.8%, two studies) followed by Africa (27.3%; 95% CI = 17.8–39.5%, PI = 1.7–89.1%, 33 studies), and Asia (12.2%; 95% CI = 4.5–29.2%, PI = 0.5–78.5%, 11 studies). Case-control studies reported the highest proportion (31.1%; 95% CI = 19.0–46.4%, PI = 2.9–87.2%, 19 studies), followed by cross-sectional (18.3%; 95% CI = 9.5–32.4%, PI = 0.6–89.5%, n = 25) and cohort studies (15.5%; 95% CI = 0.0–100.0%, PI = 1.1–75.9%, two studies). The pooled proportion was highest among studies based on PCR detection (28.2%; 95% CI = 12.0–53.1%, PI = 0.6–96.5%, 18 studies), followed by those using culture-based detection (19.9%; 95% CI = 13.3–28.8%, PI = 2.1–74.8%, 28 studies) (**Table 2**).

Africa showed the highest pooled proportion of *Salmonella* spp. (2.7%; 95% CI = 2.0–3.6%, PI = 0.4–14.2%, 41 studies), followed by Asia (2.5%; 95% CI = 1.2–5.2%, PI = 0.2–21.9%, 12 studies). Cohort study designs reported higher proportions (34.1%; 95% CI = 21.4–49.7%, one study) than cross-sectional (3.0%; 95% CI = 2.2–4.1%, PI = 0.7–12.8%, 30 studies) and case-control studies (1.9%; 95% CI = 1.2–3.0%, PI = 0.3–12.3%, 22 studies). The estimated pooled proportion based on PCR detection (2.7%; 95% CI = 1.1–6.7%, PI = 0.2–24.1%, nine studies) was similar to culture-based detection (2.6%; 95% CI = 1.9–3.5%, PI = 0.4–14.4%, 44 studies) (**Table 2**).

The highest proportion for *Shigella* spp. was observed in Asia (13.6%; 95% CI = 8.0–22.2%, PI = 2.0–55.1%, 14 studies) followed by Africa (7.9%; 95% CI = 5.7–10.7%, PI = 1.0–41.9%, 43 studies) and America (4.3%; 95% CI = 0.0–98.1%, PI = 0.0–96.1%, three studies). Cohort studies reported the highest proportion (15.6%; 95% CI = 3.8–46.4%, PI = 0.4–89.0%, eight studies), followed by those with case-control (11.5%; 95% CI = 6.7–19.0%, PI = 1.4–54.5%, 16 studies) and cross-sectional designs (6.8%; 95% CI = 4.9–9.3%, PI = 1.0–33.7%, 36 studies). The estimated pooled proportion based on PCR detection (22.4%; 95% CI = 17.0–28.8%, PI = 6.6–54.2%, 20 studies) consistently exceeded those of culture-based detection methods (5.3%; 95% CI = 3.9–7.2%, PI = 0.8–27.8%, 40 studies). Community-based samples (13.6%; 95% CI = 6.5–26.4%, PI = 0.7–77.4%, 16 studies) reported higher pooled proportions than hospital-based samples (7.5%; 95% CI = 5.6–10.0%, PI = 1.1–36.7%, 44 studies) (**Table 2**).

For DEC, we observed an apparent peak in 2014 (77.9%, 95% CI = 0.1–100.0, PI = 28.6–96.9%). However, this trend was driven by only two PCR-based studies and should therefore be interpreted with caution. Other pathogens showed no consistent temporal trends; for instance, in the case of *Campylobacter* spp., estimates were generally stable, though subgroups with few studies (*e.g.* 2021, two studies) produced wide prediction intervals. Estimates for *Salmonella* spp. remained low across time and countries, while *Shigella* spp. showed some variability, including a higher estimate in 2020 (20.7%; 95% CI = 11.7–34.1%; PI = 3.8–63.7%; 10 studies), reflecting the influence of study numbers and distribution (Tables S7 and S8 in the **Online Supplementary Document**).

### Multiple metaregression and sources of heterogeneity

We conducted meta-regression analyses to explore potential moderators of heterogeneity across studies for *Campylobacter* spp., DEC, *Salmonella* spp., and *Shigella* spp. Univariable and multivariable models were fitted, and the explanatory power of each moderator was assessed using R^2^ values.

In the univariable analyses for *Campylobacter* spp., continent, study design, source population, study duration, and sample size did not significantly explain between-study variation. Diagnostic method (PCR *vs*. culture) was a strong predictor (*β* = 1.48, *P* < 0.01), and year of study also showed a modest, but statistically significant effect (*β* = 0.09, *P* = 0.01). In the multivariable model, both diagnostic method and year of study remained significant predictors (PCR: *β* = 1.32, *P* < 0.01; year: *β* = 0.06, *P* = 0.03) (Table S9 in the **Online Supplementary Document**). Regarding heterogeneity, the diagnostic method alone explained 34.5% of the between-study heterogeneity, while the year of study alone did not. Together, the diagnostic method and year of study explained 49.4% of the between-study heterogeneity. An interaction model (diagnosis × year) further improved explanatory power, raising the explained heterogeneity to 59.1%.

For DEC, none of the examined moderators showed significant associations in univariable or multivariable models (all *P* > 0.05). The pseudo-R^2^ values for all variables were close to zero, indicating that heterogeneity in DEC estimates could not be explained by the tested study-level covariates.

In the univariable meta-regressions for *Salmonella* spp., study design, study duration, and sample size were significant predictors of heterogeneity. Cohort studies reported higher estimates compared to case-control studies (*β* = 3.24, *P* < 0.01), while we found no significant association for cross-sectional studies (*β* = 0.44, *P* = 0.09). Longer study duration was associated with lower estimates (*β* = –0.85, *P* < 0.01), as were larger sample sizes (*β* = –0.93, *P* < 0.01). In the multivariable model including study design, study duration, and sample size, cohort studies remained significant (*β* = 2.42, *P* < 0.01), while study duration and sample size did not (Table S9 in the **Online Supplementary Document**). The full model explained 66.8% of the heterogeneity. Interaction analyses showed that combining study design × study duration (pseudo-R^2^ = 69.3%), study design × sample size (pseudo-R^2^ = 67.6%), and study duration × sample size (pseudo-R^2^ = 67.2%) further increased explanatory power.

The univariable models for *Shigella* spp. identified diagnostic method (PCR *vs*. culture) as a strong predictor (*β* = 1.62, *P* < 0.01), while study year also reached a modest, but statistically significant effect (*β* = 0.10, *P* < 0.01). Study design and source population showed no or marginal associations (cross-sectional: *β* = –0.58, *P* = 0.09; hospital: *β* = –0.69, *P* = 0.04). The diagnostic method (*β* = 1.61, *P* < 0.01) and year (*β* = 0.06, *P* = 0.01) remained significant in the multivariable model (Table S9 in the **Online Supplementary Document**). This multivariable model explained 69.6% of heterogeneity. Including an interaction between diagnostic method and study year further improved the explanatory power, raising pseudo-R^2^ to 71.3%.

### Sensitivity analysis

Across all four organisms, sequential omission of individual data sets did not materially alter the pooled values or the width of their 95% CIs, thereby reinforcing the robustness of the results derived from generalised linear mixed models. For *Campylobacter* spp., the summary estimates consistently ranged from 8.1% to 9.0% with overlapping CIs, and no study exerted undue influence on the overall effect size. In the case of DEC, the pooled value was 23.0% (95% CI = 15.4–32.8), and the estimates varied between 21.7% and 24.6%, confirming stability. A similar pattern was evident for *Salmonella* spp., where the pooled effect was 2.6% (95% CI = 2.0–3.4) and omission of individual studies shifted the estimates only slightly (2.5–2.8%). Likewise, for *Shigella* spp., the pooled effect was 8.8% (95% CI = 6.6–11.7), with the leave-one-out results consistently ranging from 8.5% to 9.4%. Overall, the sensitivity analyses confirmed that the pooled effect sizes for all organisms were robust and not driven by any single influential study, which ultimately enhanced confidence in the reliability of our findings, despite the observed high heterogeneity (Figures S2–5 in the **Online Supplementary Document**).

### Bias diagnostics across multiple methods

Egger’s regression test indicated significant funnel plot asymmetry for all four pathogens (*P* < 0.01 for *Campylobacter* spp., DEC, *Salmonella* spp., and *Shigella* spp.). Rank correlation tests supported this finding for *Salmonella* spp. (*P* = 0.01) and *Campylobacter* spp. (*P* = 0.05), but were borderline for DEC (*P* = 0.07) and non-significant for *Shigella* spp. (*P* = 0.17). Doi plots with LFK indices suggested major asymmetry for *Campylobacter* spp. (–5.3), DEC (–2.3), and *Shigella* spp. (–4.5), and moderate asymmetry for *Salmonella* spp. (–1.8). Taken together, these results point to evidence of small-study effects and substantial between-study heterogeneity, rather than systematic publication bias (Figure S6 and Table S10 in the **Online Supplementary Document**).

## DISCUSSION

Enteropathogenic diarrhoea is a major cause of childhood diarrhoea in LMICs. In our review, DEC was the most frequently detected pathogen, followed by *Shigella* spp., *Campylobacter* spp., and *Salmonella* spp; however, the proportion of diarrhoeal stool samples positive for each pathogen varied between studies, regions, and by detection method. This variability presents a challenge for global burden estimation and undermines the comparability of surveillance efforts across different settings. While regional differences in pathogen proportion in diarrhoeal stool samples suggest the need for country-specific surveys and interventions, they may also reflect disparities in the number of research publications and diagnostic capacity. This highlights the need for investment in both diagnostic infrastructure and harmonisation of detection protocols, particularly in LMICs, where laboratory capacity is limited. Encouragingly, an increasing number of studies since 2018 suggest that pathogen detection efforts are improving in many regions.

*Campylobacter* spp. is particularly concerning for childhood diarrhoea in both developed and developing nations [110]. In LMICs, the burden of this bacterium is driven by poor sanitation, unsafe water, and close animal contact, while in developed countries, it remains problematic due to industrialised food systems, poultry contamination, and antimicrobial resistance, making it a global public health concern. In African countries, reported proportions of stool samples positive for *Campylobacter* spp. among children under five with diarrhoea varied considerably. For instance, Guinea-Bissau (one study) reported a proportion of 51.8%, followed by The Gambia (39.4%; one study) and Tanzania (6.8%; four studies). These findings may reflect the influence of faecal contamination in piped water and the lack of sanitary toilets, which are key predisposing factors for childhood diarrhoea attributed to *Campylobacter* spp. [108]. Poor sanitary conditions in primary schools have also been shown to contribute to diarrhoeal diseases among school-aged children in African countries [111]. To reduce the burden, we emphasise improving drinking water, sanitation, and handwashing practices with soap [112], alongside strengthening laboratory capacity, standardising diagnostic algorithms (*e.g.* culture with PCR confirmation or typing where feasible), and transparent reporting of diagnostic methods to improve comparability across future studies. Proportions were consistently higher in PCR-based studies compared to culture, highlighting test-related variability and contributing to wide prediction intervals.

We identified DEC as a commonly implicated organism causing diarrhoea among children under five years old [78,103]. There was wide variation in the proportion of stool samples positive for DEC across studies that was only partly explained by diagnostic differences, with PCR detecting higher values than culture. Remaining variability likely reflects merging of DEC pathotypes, inconsistencies in laboratory procedures, and unmeasured contextual factors such as climate, seasonality, or water quality. Because individual DEC pathotypes were inconsistently reported, we pooled them as a group, which may obscure differences between strains but ensured consistency across studies. Standardising pathogen categorisation in future investigations may benefit future analyses. Surveillance should be emphasised, including strain-specific differentiation and genomic sequencing, which could enhance organism detection and targeted interventions, although this may not be practical for many LMICs due to limited laboratory and public health infrastructure. Moreover, we recommend safe food handling, avoiding undercooked meat and unpasteurised dairy product consumption, and maintaining hand hygiene practices to prevent DEC-induced childhood diarrhoea [113].

We observed a significant proportion of *Salmonella*-positive diarrhoeal stool samples in LMICs. Multiple studies reported similar detection rates of *Salmonella* spp. among affected children in African countries, likely influenced by geographic location, water facilities, and personal hygiene practices [77,79]. In Asia, Bangladesh had the highest reported burden of *Salmonella* spp. in diarrhoea-affected child stool samples, potentially due to unsafe water, inadequate sanitation, poor food handling, and childhood malnutrition [114]. Unlike *Campylobacter* spp. and *Shigella* spp., differences between PCR and culture were modest, but PIs indicated that study-level estimates could still vary widely. The lower proportion of diarrhoeal stool samples positive for *Salmonella* spp. among children under five with diarrhoea observed in hospital-based studies may reflect selection bias, possibly due to over-representation of viral cases (*e.g.* rotavirus), younger children, or prior antibiotic use before hospitalisation, and warrants further investigation. Across all pathogens, hospital- *vs*. community-based sampling could not be consistently disentangled, which may have further contributed to between-study heterogeneity. However, appropriate cooking of meat and eggs, prevention of cross-contamination during food preparation, and safe handling of animals may reduce the transmission of *Salmonella* spp. [115,116]. Importantly, all these preventive measures require behavioural change and a community education programme focussing on the rural and peri-urban setting of LMICs.

*Shigella* spp., another leading cause of childhood diarrhoea [117], disproportionately affects children due to their undeveloped immune system and limited understanding and practice of personal hygiene [118]. In our analysis, the PCR-based diagnostic method was the strongest contributor to variability in the proportion of *Shigella*-positive diarrhoeal stool samples. This pattern likely reflects the greater sensitivity of molecular assays, but also possible cross-detection of related *E. coli* strains, whereas culture is more specific but underestimates cases. Considering individual countries, Burkina Faso reported proportions varied widely, reflecting the variability across studies [51,54,119]. Similarly, substantial differences were observed in Guinea-Bissau and India, where confidence intervals indicated high variation across studies [96,120], highlighting local variability in pathogen burden. In America, two studies included in this review reported similar proportions of *Shigella* spp. among children with diarrhoea. Therefore, improving sanitation, increasing access to clean water, and practicing proper handwashing, especially after toilet use, may prevent this infection among children below five [113]. In addition, growing antimicrobial resistance in *Shigella*, especially in Asia and Africa, underscores the need to integrate enteric pathogen data into national and global AMR surveillance programmes.

Heterogeneity remained substantial across all pathogens (*I*^2 ^> 90%), but the meta-regression identified several key contributors. Diagnostic method and study year explained nearly half the variability for *Campylobacter* spp. and more than two-thirds for *Shigella* spp., while study design and sample size were the strongest drivers for *Salmonella* spp. In contrast, heterogeneity in DEC estimates was not well explained by the tested moderators, suggesting that unmeasured factors such as seasonal patterns, water quality, or case ascertainment practices may be important. Such heterogeneity is expected given differences in study populations and methodologies. Beyond diagnostic method and study design, residual variability may also reflect differences in the types of children included, seasonal patterns, and case detection practices. Interaction effects were explored where univariable analyses indicated key drivers like diagnostic method with year for *Campylobacter* spp. and *Shigella* spp., and study design with sample size or duration for *Salmonella* spp. to better capture how combinations of methodological factors contributed to between-study heterogeneity. Pooling across different study designs can be challenging, because cross-sectional, case-control, and cohort studies carry distinct sources of bias. However, by harmonising data extraction to include only diarrhoeal cases under five years of age and their first stool sample tested for pathogens, we ensured conceptual equivalence across study types. Sensitivity analyses confirmed that no single design disproportionately influenced the pooled estimates, justifying this methodological decision. Nonetheless, residual differences in sampling frames and study implementation may still contribute to heterogeneity, which should be considered when interpreting results. These findings highlight that diagnostic approaches and study characteristics do not operate independently; rather, their effects vary over time and in combination, contributing to the observed variability across studies. Accordingly, we emphasised both statistical measures (*I*^2^, *τ*^2^) and substantive sources of heterogeneity [121]. These differences underscore that pooled proportions should be interpreted with caution, and prediction intervals provide a more realistic range of expected study-level results. Standardising diagnostic methods and improving reporting would help reduce heterogeneity and improve comparability in future research.

This study has several limitations. Dependence on published, English-language studies may have introduced biases, and geographic representation was uneven, with some countries represented by only a single study and large regions lacking data. Although we harmonised extraction across study designs to ensure conceptual equivalence, pooling across designs, settings, and diagnostic methods may still have introduced residual bias and contributed to the substantial heterogeneity observed. Leave-one-out analyses confirmed robustness of pooled estimates, yet residual variation remained high despite subgroup analyses. LFK indices suggested major asymmetries for all pathogens, more likely attributable to heterogeneity and extreme study-level estimates than to systematic reporting bias. It should also be noted that Egger’s regression test is highly sensitive to heterogeneity, and significant results in this context may reflect variance inflation, rather than true publication bias. Seasonal variation and case detection practices were not consistently reported, limiting our ability to account for these factors. We also acknowledge that while we performed subgroup analyses stratified by diagnostic method (culture *vs*. PCR), we did not apply formal test-performance calibration using sensitivity/specificity estimates, as context-specific values for paediatric populations were not consistently available in LMIC settings. We did not apply a formal hierarchy of evidence approach or restrict analyses by study design, given harmonisation and sensitivity analyses demonstrated no disproportionate influence of any single design. Future reviews with larger numbers of longitudinal studies may explore this further. Most studies tested for multiple pathogens, so pathogen-positive proportions are not mutually exclusive. Finally, while quality varied, we retained all eligible studies to ensure representation from under-studied regions, potentially introducing variability. Nevertheless, we note that the overall evidence is of moderate strength and remains policy-relevant for guiding surveillance and WASH interventions in LMICs.

## CONCLUSIONS

Our findings underscore the substantial burden of childhood diarrhoea in LMICs attributable to *Campylobacter* spp., DEC, *Salmonella* spp., and *Shigella* spp. While differences in study design, sample size, and diagnostic methods explained some heterogeneity, considerable residual variation between studies and countries remains. Pooled values should, therefore, be interpreted with caution, as PIs highlight the wide range of possible study-level results. Despite this uncertainty, the estimates remain policy-relevant, as they provide a synthesised overview of pathogen-specific burdens in LMICs that can inform laboratory capacity planning, standardisation of diagnostic algorithms, and water, sanitation, and hygiene-related interventions. Future research should prioritise understudied regions and incorporate improved reporting of diagnostic methods to strengthen comparability and guide more targeted surveillance and control strategies.

## Additional material


Online Supplementary Document
