# Supplementary material for: Childhood diarrhoea attributed to enteropathogenic bacteria in low- and middle-income countries: a systematic review and meta-analysis
Source: J Glob Health. 2025 Nov 28;15:04350. doi: 10.7189/jogh.15.04350 (PMC12659801; doi:10.7189/jogh.15.04350)
Supplement: Online Supplementary Document [file jogh-15-04350-s001.pdf]

Supplement to: Sayeed MA, Colquhoun S, Thottunkal S, McLure A, Richardson A, Lal A, Rahaman MR. Childhood diarrhea attributed to enteropathogenic bacteria in low- and middle-income countries: a systematic review and meta-analysis. J Glob Health. 2025;15:04350.

Table S 1. Systematic review inclusion and exclusion criteria. 2
Table S 2. PRISMA-S–compliant search strategies and dates of initial and updated searches........ 3
Table S 3. Reviewer agreement during screening of studies for inclusion in the systematic review. 5
Table S 4. Quality assessment of the reported articles that used in meta-analysis using Joanna Briggs Institute (JBI) tools 6
Table S 5. Data extraction proforma used for systematic review of enteropathogenic bacteria in childhood diarrhea 9
Table S 6. Pooled proportion of DEC pathotypes in childhood diarrhea........ 16
Table S 7. Country specific pooled proportion of enteric pathogens........ 17
Table S 8. Year specific pooled proportion of enteric pathogens........ 18
Table S 9. Meta-regression of *Campylobacter* spp., DEC, *Salmonella* spp., and *Shigella* spp. among diarrhea affected children in LMIC's........ 19
Table S 10. Results of Egger's regression test, rank correlation test, and DOI plot (LFK index) for assessing potential publication bias and small-study effects in the meta-analysis of enteropathogenic bacteria........ 27

Complete bibliography 1. Bibliography of all 71 articles [1-71]........ 10

Figure S 1. Flow diagram of the statistical analysis plan........ 10
Figure S 2. Leave-one-out (LOO) sensitivity analysis for *Campylobacter* spp........ 22
Figure S 3. Leave-one-out (LOO) sensitivity analysis for DEC........ 23
Figure S 4. Leave-one-out (LOO) sensitivity analysis for *Salmonella* spp. 24
Figure S 5. Leave-one-out (LOO) sensitivity analysis for *Shigella* spp. 25
Figure S 6. Doi plot of studies (n=84) included in the meta-analysis, with z-score on the y-axes and proportion for each pathogen on the x-axes: A. *Campylobacter* spp.; B. DEC; C. *Salmonella* spp.; D. *Shigella* spp........ 26

Table S 1. Systematic review inclusion and exclusion criteria.

Eligibility Criteria Domain	Inclusion Criteria	Exclusion Criteria
**Population**	Children under five years of age residing in low- and middle-income countries	Children under five years of age residing in high income countries
**Exposure**	Childhood diarrhea caused by enteric pathogens, including but not limited to *Campylobacter* spp., *Salmonella* spp., *E. coli*, and *Shigella* spp. infections in children aged <5 years old	Studies not focusing on *Campylobacter spp., Salmonella spp., Escherichia coli (E. coli)*, and *Shigella spp.* infections in children
**Outcome**	Childhood diarrhea cases due to enteropathogenic bacterial infection	Diarrhea over 5 years of age
**Study design**	Original research articles from:<br>• Cross sectional studies<br>• Cohort studies<br>• Case control studies<br>• Experimental studies<br><br>Available in full text, published in English language.	Conference abstracts or proceedings.<br>Case reports.<br>Case series<br>Letters to the Editor<br>Grey literature<br>Reviews, including systematic reviews.
**Study period**	Articles published during 2000-2025	Article published before 2000

Table S 2. PRISMA-S–compliant search strategies and dates of initial and updated searches

Database / Source	Date of initial search	Date of last search	Search strategy
**PubMed**	April 15, 2024	February 10, 2025	((“child*”[All Fields] OR “infant”[MeSH Terms] OR “infant”[All Fields] OR “infants”[All Fields]) AND (“diarrhea”[MeSH Terms] OR “diarrhea”[All Fields] OR “diarrhoea”[All Fields] OR “diarr*”[All Fields] OR “Diarrhoeal disease”[All Fields] OR “Escherichia coli”[All Fields] OR “enterotoxigenic”[All Fields] OR “e coli”[All Fields] OR “campylobac*”[All Fields] OR “salmonella”[MeSH Terms] OR “salmonella”[All Fields] OR “shigell*”[All Fields]) AND (“cases”[All Fields] OR “incidence”[MeSH Terms] OR “incidence”[All Fields] OR “prevalence”[MeSH Terms] OR “prevalence”[All Fields]) AND “food*”[All Fields] AND (“epidemiology”[MeSH Subheading] OR “observational”[All Fields] OR “intervention*”[All Fields] OR “case-control”[All Fields] OR “cross sectional stud*”[All Fields] OR “cohort”[All Fields]) AND (“developing countr*”[All Fields] OR “low and middle income countr*”[All Fields] OR “third world countr*”[All Fields] OR “least developed countr*”[All Fields]) AND (“risk”[MeSH Terms] OR “exposure*”[All Fields] OR “source*”[All Fields] OR “determinant*”[All Fields]) AND (“2000/01/01”[Date - Publication] : “2024/04/15”[Date - Publication]))
**Embase**	April 15, 2024	February 10, 2025	((((((((child* OR infant) AND diarrhea OR diarr* OR "Diarrhoeal disease" OR "Escherichia coli" OR enterotoxigenic OR "E. coli" OR campylobac* OR salmonella OR shigell*) AND (cases OR incidence OR prevalence)) AND food* AND (epidemiology OR observational OR intervention OR case-control OR "cross-sectional stud*" OR cohort)) AND ("Developing countr*" OR "low and middle income countr*" OR "third world countr*" OR "least developed countr*")) AND (risk OR exposure OR source OR determinant*)).mp. [mp=title, abstract, full text, caption text]) LIMIT 1 to yr="2000 - 2024")
**Scopus**	April 15, 2024	February 10, 2025	TITLE-ABS-KEY((child* OR infant) AND (diarrhea OR diarr* OR "Diarrhoeal disease" OR "Escherichia coli" OR enterotoxigenic OR "E. coli" OR campylobac* OR salmonella OR shigell*) AND (cases OR incidence OR prevalence) AND food* AND (epidemiology OR observational OR intervention OR case-control OR "cross-sectional stud*" OR cohort) AND ("Developing countr*" OR "low and middle income countr*" OR "third world countr*" OR "least developed countr*") AND (risk OR exposure OR source OR determinant*))

**Web of Science**	April 15, 2024	February 10, 2025	ALL=(((child* OR infant) AND (diarrhea OR diarr* OR "Diarrhoeal disease" OR "Escherichia coli" OR enterotoxigenic OR "E. coli" OR campylobac* OR salmonella OR shigell*) AND (cases OR incidence OR prevalence) AND food* AND (epidemiology OR observational OR intervention OR case-control OR "cross-sectional stud*" OR cohort) AND ("Developing countr*" OR "low and middle income countr*" OR "third world countr*" OR "least developed countr*") AND (risk OR exposure OR source OR determinant*)))
**ProQuest**	April 15, 2024	February 10, 2025	[STRICT] ((child* OR infant) AND (diarrhea OR diarr* OR "Diarrhoeal disease" OR "Escherichia coli" OR Enterotoxigenic OR "E. coli" OR campylobac* OR Salmonella OR Shigell*) AND (cases OR incidence OR prevalence) AND (food*) AND (epidemiology OR observational OR intervention OR case-control OR "cross-sectional stud*" OR cohort) AND ("Developing countr*" OR "low and middle income countr*" OR "third world countr*" OR "least developed countr*") AND (risk OR exposure OR source OR determinant*))
**Google Scholar**	April 15, 2024	February 10, 2025	((child* OR infant) AND (diarrhea OR diarr* OR "Diarrhoeal disease" OR "Escherichia coli" OR enterotoxigenic OR "E. coli" OR campylobac* OR salmonella OR shigell*) AND (cases OR incidence OR prevalence) AND food* AND (epidemiology OR observational OR intervention OR case-control OR "cross-sectional stud*" OR cohort) AND ("Developing countr*" OR "low and middle income countr*" OR "third world countr*" OR "least developed countr*") AND (risk OR exposure OR source OR determinant*))

Table S 3. Reviewer agreement during screening of studies for inclusion in the systematic review.

Category of identification of studies	Screening Stage	Records Screened	Agreements (n)	Disagreements (n)	Agreement (%)	Kappa
**Database search**	Title and Abstract	4,252	3,912	340	92.0%	0.84
	Full-text	128	121	7	94.5%	0.89
**Forward and backward search**	Title and Abstract	4,057	3,703	354	91.2%	0.82
	Full-text	114	108	6	95.0%	0.90

Table S 4. Quality assessment of the reported articles that used in meta-analysis using Joanna Briggs Institute (JBI) tools

Paper/Author	1. Was the sample frame appropriate to address the target population?	2. Were study participants sampled in an appropriate way?	3. Was the sample size adequate?	4. Were the study subjects and the setting described in detail?	5. Was the data analysis conducted with sufficient coverage of the identified sample?	6. Were valid methods used for the identification of the condition?	7. Was the condition measured in a standard, reliable way for all participants?	8. Was there appropriate statistical analysis?	9. Was the response rate adequate/was the low response rate managed appropriately?	Overall score	Overall appraisal
**Qadri, F., et al. (2000)**	no	yes	no	yes	yes	yes	yes	yes	yes	0.77	strong
**Isenbarger, D.W., et al. (2001)**	yes	yes	yes	yes	yes	yes	yes	yes	yes	1	V strong
**Vargas, M., et al. (2004)**	yes	yes	yes	yes	yes	yes	yes	yes	yes	1	V strong
**Nguyen, T.V., et al. (2006)**	yes	yes	no	yes	yes	yes	yes	yes	yes	0.88	strong
**Mandomando, I., et al. (2007)**	yes	yes	yes	yes	yes	yes	yes	yes	yes	1	V strong
**Moyo, S.J., et al. (2007)**	no	yes	no	yes	yes	yes	yes	yes	yes	0.77	strong
**Hien, B.T.T., et al. (2008)**	no	yes	no	yes	yes	yes	yes	yes	yes	0.77	strong
**Beatty, M.E., et al. (2009)**	yes	yes	yes	yes	yes	yes	yes	yes	yes	1	V strong
**Rivera, F.P., et al. (2010)**	yes	yes	yes	yes	yes	yes	yes	yes	yes	1	V strong
**Debas, G., et al. (2011)**	no	yes	no	yes	yes	yes	yes	yes	yes	0.77	strong
**Moyo, S.J., et al. (2011)**	no	yes	no	yes	yes	yes	yes	yes	yes	0.77	strong
**Nitiema, L.W., et al. (2011)**	yes	yes	yes	yes	yes	yes	yes	yes	yes	1	V strong
**Ansari, S., et al. (2012)**	yes	yes	yes	yes	yes	yes	yes	yes	yes	1	V strong
**Randremanana, R., et al. (2012)**	yes	yes	yes	yes	yes	yes	yes	yes	yes	1	V strong
**Bonkoungou, I.J.O., et al. (2013)**	no	yes	no	yes	yes	yes	yes	yes	yes	0.77	strong
**Lengerh, A., et al. (2013)**	no	yes	no	yes	yes	yes	yes	yes	yes	0.77	strong
**Mason, J., et al. (2013)**	yes	yes	yes	yes	yes	yes	yes	yes	yes	1	V strong
**Sambe-Ba, B., et al. (2013)**	no	yes	no	yes	yes	yes	yes	yes	yes	0.77	strong
**Thompson, C.N., et al. (2013)**	yes	yes	yes	yes	yes	yes	yes	yes	yes	1	V strong
**Beyene, G. and H. Tasew (2014)**	yes	yes	yes	yes	yes	yes	yes	yes	yes	1	V strong
**Deogratias, A., et al. (2014)**	no	yes	no	yes	yes	yes	yes	yes	yes	0.77	strong
**Kabayiza, J., et al. (2014)**	yes	yes	yes	yes	yes	yes	yes	yes	yes	1	V strong
**Mulatu, G., et al. (2014)**	no	yes	no	yes	yes	yes	yes	yes	yes	0.77	strong
**Tafa, B., et al. (2014)**	no	yes	no	yes	yes	yes	yes	yes	yes	0.77	strong

**Admassu, M., et al. (2015)**	yes	yes	yes	yes	yes	yes	yes	yes	yes	1	V strong
**Benmessaoud, R., et al. (2015)**	no	yes	no	yes	yes	yes	yes	yes	yes	0.77	strong
**Gebrekidan Kahsay, A. and Z. Teklemariam (2015)**	no	no	no	yes	yes	yes	yes	yes	yes	0.66	average
**Mamuye, Y., et al. (2015)**	no	yes	no	yes	yes	yes	yes	yes	yes	0.77	strong
**Nhampossa, T., et al. (2015)**	yes	yes	yes	yes	yes	yes	yes	yes	yes	1	V strong
**Saeed, A., et al. (2015)**	yes	yes	yes	yes	yes	yes	yes	yes	yes	1	V strong
**Breurec, S., et al. (2016)**	yes	yes	yes	yes	yes	yes	yes	yes	yes	1	V strong
**Shah, M.M., et al. (2016)**	yes	yes	yes	yes	yes	yes	yes	yes	yes	1	V strong
**Andersson, M., et al. (2017)**	yes	yes	yes	yes	yes	yes	yes	yes	yes	1	V strong
**Ashie, G.K., et al. (2017)**	yes	yes	yes	yes	yes	yes	yes	yes	yes	1	V strong
**Chiyangi, H., et al. (2017)**	no	yes	no	yes	yes	yes	yes	yes	yes	0.77	strong
**Gosselin, K.B., et al. (2017)**	no	no	no	yes	yes	yes	yes	yes	yes	0.66	average
**Ikumapayi, U.N., et al. (2017)**	yes	yes	yes	yes	yes	yes	yes	yes	yes	1	V strong
**Shah, M.M., et al. (2017)**	yes	yes	yes	yes	yes	yes	yes	yes	yes	1	V strong
**Abebe, W., et al. (2018)**	no	yes	no	yes	yes	yes	yes	yes	yes	0.77	strong
**Adam, M.A., et al. (2018)**	no	yes	no	yes	yes	yes	yes	yes	yes	0.77	strong
**Ameya, G., et al. (2018)**	no	yes	no	yes	yes	yes	yes	yes	yes	0.77	strong
**Francois, R., et al. (2018)**	yes	yes	yes	yes	yes	yes	yes	yes	yes	1	V strong
**GebreSilasie, et al. (2018)**	no	yes	no	yes	yes	yes	yes	yes	yes	0.77	strong
**Vubil, D., et al. (2018)**	yes	yes	yes	yes	yes	yes	yes	yes	yes	1	V strong
**Acácio, S., et al. (2019)**	yes	yes	yes	yes	yes	yes	yes	yes	yes	1	V strong
**Assefa, A. and M. Girma (2019)**	yes	yes	yes	yes	yes	yes	yes	yes	yes	1	V strong
**Jahan, Y., et al. (2019)**	yes	yes	yes	yes	yes	yes	yes	yes	yes	1	V strong
**Mohakud, N., et al. (2019)**	no	yes	no	yes	yes	yes	yes	yes	yes	0.77	strong
**Berendes, D., et al. (2020)**	yes	yes	yes	yes	yes	yes	yes	yes	yes	1	V strong
**Borkakoty, B., et al. (2020)**	yes	yes	yes	yes	yes	yes	yes	yes	yes	1	V strong
**Fagerli, K., et al. (2020)**	yes	yes	yes	yes	yes	yes	yes	yes	yes	1	V strong
**Garrine, M., et al. (2020)**	yes	no	yes	yes	yes	yes	yes	yes	yes	0.88	strong
**Jain, P., et al. (2020)**	yes	yes	yes	yes	yes	yes	yes	yes	yes	1	V strong

**McQuade, E.T.R., et al. (2020)**	yes	yes	yes	yes	yes	yes	yes	yes	yes	1	V strong
**Singh, M.K., et al. (2020)**	yes	yes	yes	yes	yes	yes	yes	yes	yes	1	V strong
**Tosisa, W., et al. (2020)**	no	yes	no	yes	yes	yes	yes	yes	yes	0.77	strong
**Kasumba, I.N., et al. (2021)**	yes	yes	yes	yes	yes	yes	yes	yes	yes	1	V strong
**Mero, S., et al. (2021)**	yes	yes	yes	yes	yes	yes	yes	yes	yes	1	V strong
**Uddin, M.S., et al. (2021)**	yes	yes	yes	yes	yes	yes	yes	yes	yes	1	V strong
**Zachariah, O.H., et al. (2021)**	no	yes	no	yes	yes	yes	yes	yes	yes	0.77	strong
**Behailu, Y., et al. (2022)**	no	yes	no	yes	yes	yes	yes	yes	yes	0.77	strong
**Leting, S.K., et al. (2022)**	no	yes	no	yes	yes	yes	yes	yes	yes	0.77	strong
**Nigusu, Y., et al. (2022)**	no	yes	no	yes	yes	yes	yes	yes	yes	0.77	strong
**Shrestha, S.K., et al. (2022)**	yes	yes	yes	yes	yes	yes	yes	yes	yes	1	V strong
**Akinlabi, O.C., et al. (2023)**	yes	yes	yes	yes	yes	yes	yes	yes	yes	1	V strong
**Belina, D., et al. (2023)**	no	yes	no	yes	yes	yes	yes	yes	yes	0.77	strong
**Berendes, D.M., et al. (2023)**	yes	yes	yes	yes	yes	yes	yes	yes	yes	1	V strong
**Dessale, M., et al. (2023)**	no	yes	no	yes	yes	yes	yes	yes	yes	0.77	strong
**Diaz, J.N., et al. (2023)**	no	yes	no	yes	yes	yes	yes	yes	yes	0.77	strong
**Mwape, K., et al. (2023)**	yes	yes	yes	yes	yes	yes	yes	yes	yes	1	V strong
**Worku et al., 2024**	yes	yes	yes	yes	yes	yes	yes	yes	yes	1	V strong

*"Yes" = 1 and "No" = 0, following a binary scoring scale. A mean score of 1 indicates Very Strong (V. strong), while values from 0.75 to <1 indicate Strong and <0.50 to <0.75 indicate average.

Table S 5. Data extraction proforma used for systematic review of enteropathogenic bacteria in childhood diarrhea

**General Information**																
**Sl. No**	Title	Citation	Journal	Year	DOI /Link	Study design	Primary outcome	Secondary outcome (if any)	Study location	Continent	Economic status (WB 2023)	Year of study conducted	Duration of study	Source population	Diagnostic methods	Age of participants
***E. coli* / DEC**																
**Any DEC reported (Yes/No)**	Total diarrheal samples tested for *E. coli*		Total diarrheal samples positive for *E. coli* (DEC)		Pathotype(s) reported (EPEC, ETEC, EAEC, STEC, etc.)		EPEC positive		ETEC positive		EAEC positive		EIEC positive		STEC positive (STEC, EHEC, VTEC)	
***Campylobacter* spp.**																
**Campylobacter spp. reported (Yes/No)**	Total diarrheal samples tested for *Campylobacter* spp.		Total diarrheal samples positive for *Campylobacter* spp.		Species/serotype(s) reported											
***Shigella* spp.**																
***Shigella* spp. reported (Yes/No)**	Total diarrheal samples tested for *Shigella* spp.		Total diarrheal samples positive for *Shigella* spp.		Species/serotype(s) reported											
***Salmonella* spp.**																
**Salmonella spp. reported (Yes/No)**	Total diarrheal samples tested for *Salmonella* spp.		Total diarrheal samples positive for *Salmonella* spp.		Species/serotype(s) reported											
**Other Study Details**																
**Definition of diarrhea**	Author conclusion															

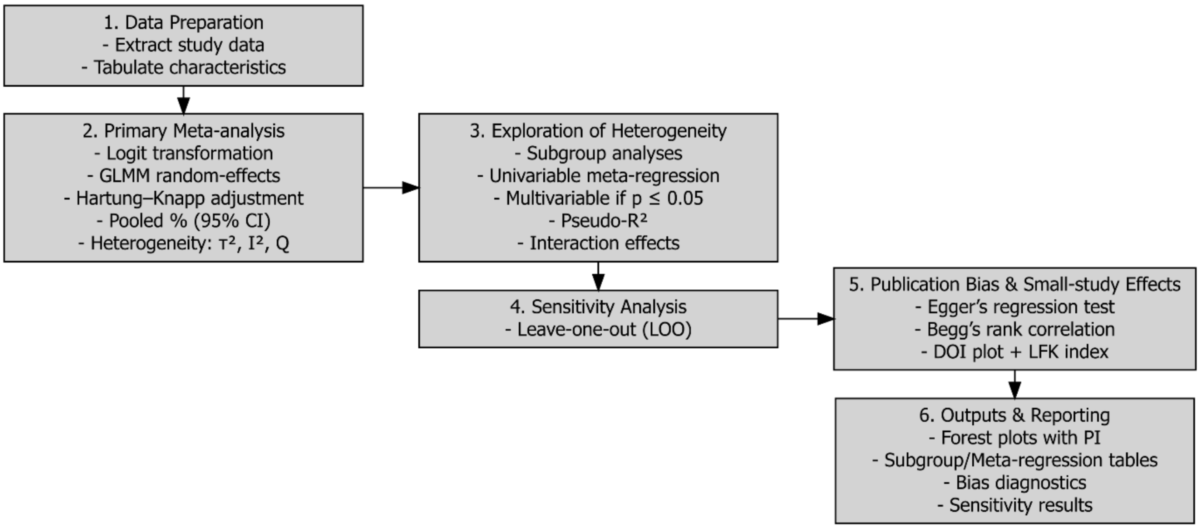

Figure S 1. Flow diagram of the statistical analysis plan.

The diagram outlines the sequential steps undertaken in the meta-analysis, beginning with data preparation and descriptive tabulation, followed by primary meta-analysis using logit-transformed generalized linear mixed models (GLMM) with Hartung–Knapp adjustment. Exploration of heterogeneity was performed through subgroup analyses and meta-regression (with pseudo-R^2 and interaction effects), and sensitivity analysis was conducted using leave-one-out (LOO) procedures. Publication bias and small-study effects were assessed using Egger's regression, Begg's rank correlation, and DOI plots with LFK index. Outputs included pooled estimates with prediction intervals, subgroup/meta-regression tables, bias diagnostics, and sensitivity results

Complete bibliography 1. Bibliography of all 71 articles [1-71]

1. Abebe W, Earsido A, Taye S, Assefa M, Eyasu A, Godebo G. Prevalence and antibiotic susceptibility patterns of Shigella and Salmonella among children aged below five years with Diarrhoea attending Nigist Eleni Mohammed memorial hospital, South Ethiopia. BMC pediatrics. 2018;18:241-.

2. Acácio S, Mandomando I, Nhampossa T, Quintó L, Vubil D, Sacoor C, et al. Risk factors for death among children 0–59 months of age with moderate-to-severe diarrhea in Manhiça district, southern Mozambique. BMC infectious diseases. 2019;19:1-14.

3. Adam MA, Wang J, Enan K, Shen H, Wang H, Hussein A, et al. Molecular Survey of Viral and Bacterial Causes of Childhood Diarrhea in Khartoum State, Sudan. Frontiers in microbiology. 2018;9:112-.

4. Admassu M, Yemane G, Kibret M, Abera B, Nibret E, Adal M. Prevalence and antibiogram of Shigella and Salmonella spp. from under five children with acute diarrhea in Bahir Dar Town. Ethiopian Journal of Science and Technology. 2015;8:27-35.

5. Akinlabi OC, El-shama QN, Dada RA, Ekpo S, Omotuyi A, Nwimo CC, et al. Epidemiology and Risk Factors for Diarrheagenic Escherichia coli Carriage among Children in Northern Ibadan, Nigeria. The American Journal of Tropical Medicine and Hygiene. 2023;109(6):1223.

6. Ameya G, Tsalla T, Getu F, Getu E. Antimicrobial susceptibility pattern, and associated factors of Salmonella and Shigella infections among under five children in Arba Minch, South Ethiopia. Annals of clinical microbiology and antimicrobials. 2018;17:1-7.

7. Andersson M, Elfving K, Shakely D, Nilsson S, Msellem MI, Trollfors B, et al. Rapid Clearance and Frequent Reinfection With Enteric Pathogens Among Children With Acute Diarrhea in Zanzibar. Clinical infectious diseases : an official publication of the Infectious Diseases Society of America. 2017;65:1371-7.

8. Ansari S, Sherchand J, Parajuli K, Mishra SK, Dahal RK, Shrestha S, et al. Bacterial etiology of acute diarrhea in children under five years of age. Journal of Nepal Health Research Council. 2012;10:218-23.

9. Ashie GK, Mutocheluh M, Owusu M, Kwofie TB, Akonor S, Narkwa PW, et al. Microbial pathogens associated with acute childhood diarrhoea in Kumasi, Ghana. BMC research notes. 2017;10:264-.

10. Assefa A, Girma M. Prevalence and antimicrobial susceptibility patterns of Salmonella and Shigella isolates among children aged below five years with diarrhea attending Robe General Hospital and Goba Referral Hospital, South East Ethiopia. Tropical diseases, travel medicine and vaccines. 2019;5:19-.

11. Beatty ME, Ochieng JB, Chege W, Kumar L, Okoth G, Shapiro RL, et al. Sporadic paediatric diarrhoeal illness in urban and rural sites in Nyanza Province, Kenya. East African medical journal. 2009;86:387-98.

12. Behailu Y, Hussen S, Alemayehu T, Mengistu M, Fenta DA. Prevalence, determinants, and antimicrobial susceptibility patterns of Campylobacter infection among under-five children with diarrhea at Governmental Hospitals in Hawassa city, Sidama, Ethiopia. A cross-sectional study. Plos one. 2022;17(5):e0266976.

13. Belina D, Gobena T, Kebede A, Chimdessa M, Hailu Y, Hald T. Occurrence of Diarrheagenic Pathogens and Their Coinfection Profiles in Diarrheic Under Five Children and Tracked Human Contacts in Urban and Rural Settings of Eastern Ethiopia. Microbiology insights. 2023;16:11786361231196527-undefined.

14. Benmessaoud R, Jroundi I, Nezha M, Moraleda C, Tligui H, Seffar M, et al. Etiology, epidemiology and clinical characteristics of acute moderate-to-severe diarrhea in children under 5 years of age hospitalized in a referral pediatric hospital in Rabat, Morocco. Journal of medical microbiology. 2015;64(1):84-92.

15. Berendes D, Capone D, Knee J, Holcomb D, Sultana S, Pickering AJ, et al. Associations between enteric pathogen carriage and height-for-age, weight-for-age and weight-for-height in children under 5 years old in urban Dhaka, Bangladesh. Epidemiology and Infection. 2020;148:10.

16. Berendes DM, Omore R, Prentice-Mott G, Fagerli K, Kim S, Nasrin D, et al. Exploring survey-based water, sanitation, and animal associations with enteric pathogen carriage: comparing results in a cohort of cases with moderate-to-severe diarrhea to those in controls in the Vaccine Impact on Diarrhea in Africa (VIDA) study, 2015–2018. Clinical Infectious Diseases. 2023;76(Supplement_1):S140-S52.

17. Beyene G, Tasew H. Prevalence of intestinal parasite, Shigella and Salmonella species among diarrheal children in Jimma health center, Jimma southwest Ethiopia: a cross sectional study. Annals of clinical microbiology and antimicrobials. 2014;13:10-.

18. Bonkoungou IJO, Haukka K, Österblad M, Hakanen AJ, Traoré AS, Barro N, et al. Bacterial and viral etiology of childhood diarrhea in Ouagadougou, Burkina Faso. BMC pediatrics. 2013;13(1):1-6.

19. Borkakoty B, Jakharia A, Sarmah MD, Hazarika R, Baruah PJ, Bora CJ, et al. Prevalence of campylobacter enteritis in children under 5 years hospitalised for diarrhoea in two cities of Northeast India. Indian journal of medical microbiology. 2020;38:32-6.

20. Breurec S, Vanel N, Bata P, Chartier L, Farra A, Favennec L, et al. Etiology and Epidemiology of Diarrhea in Hospitalized Children from Low Income Country: A Matched Case-Control Study in Central African Republic. PLoS neglected tropical diseases. 2016;10:e0004283-undefined.

21. Chiyangi H, Muma JB, Malama S, Manyahi J, Abade A, Kwenda G, et al. Identification and antimicrobial resistance patterns of bacterial enteropathogens from children aged 0–59 months at the University Teaching Hospital, Lusaka, Zambia: a prospective cross sectional study. BMC infectious diseases. 2017;17:117-.

22. Debas G, Kibret M, Biadglegne F, Abera B. Prevalence and antimicrobial susceptibility patterns of shigella species at Felege Hiwot Referral Hospital, Northwest Ethiopia. Ethiopian medical journal. 2011;49:249-56.

23. Deogratias A, Mushi MF, Paterno L, Tappe D, Seni J, Kabymera R, et al. Prevalence and determinants of Campylobacter infection among under five children with acute watery diarrhea in Mwanza, North Tanzania. Archives of public health = Archives belges de sante publique. 2014;72:17-.

24. Dessale M, Mengistu G, Mengist HM. Prevalence, antimicrobial resistance pattern, and associated factors of Salmonella and Shigella among under five diarrheic children attending public health facilities in Debre Markos town, Northwest Ethiopia. Frontiers in public health. 2023;11:1114223-undefined.

25. Diaz JN, Iannotti LL, Louis Dulience SJ, Vie S, Jiang X, Grigura V, et al. Prevalence of diarrheagenic Escherichia coli and impact on child health in Cap-Haitien, Haiti. PLOS global public health. 2023;3:e0001863-e.

26. Fagerli K, Omore R, Kim S, Ochieng J, Ayers T, Juma J, et al. Factors associated with typical enteropathogenic Escherichia coli infection among children< 5 years old with moderate-to-severe diarrhoea in rural western Kenya, 2008–2012. Epidemiology & Infection. 2020;148:e281.

27. Francois R, Yori PP, Rouhani S, Salas MS, Olortegui MP, Trigoso DR, et al. The other Campylobacters: Not innocent bystanders in endemic diarrhea and dysentery in children in low-income settings. Plos Neglected Tropical Diseases. 2018;12(2):15.

28. Garrine M, Matambisso G, Nobela NA, Vubil D, Massora S, Acácio S, et al. Low frequency of enterohemorrhagic, enteroinvasive and diffusely adherent Escherichia coli in children under 5 years in rural Mozambique: a case-control study. BMC infectious diseases. 2020;20:659-.

29. Gebrekidan Kahsay A, Teklemariam Z. Prevalence of Shigella among diarrheic children under-5 years of age attending at Mekelle health center, north Ethiopia. BMC research notes. 2015;8(1):1-4.

30. GebreSilasie YM, Kassu Desta T, Addisu Gize Y. Resistance pattern and maternal knowledge, attitude and practices of suspected Diarrheagenic Escherichia coli among children under 5 years of age in Addis Ababa, Ethiopia: cross sectional study. Antimicrobial Resistance and Infection Control. 2018;7.

31. Gosselin KB, Aboud S, McDonald CM, Moyo S, Khavari N, Manji K, et al. Etiology of diarrhea, nutritional outcomes, and novel intestinal biomarkers in Tanzanian infants. Journal of pediatric gastroenterology and nutrition. 2017;64(1):104-8.

32. Hien BTT, Scheutz F, Cam PD, Serichantalergs O, Huong TT, Thu TM, et al. Diarrheagenic Escherichia coli and Shigella Strains Isolated from Children in a Hospital Case-Control Study in Hanoi, Vietnam. Journal of clinical microbiology. 2008;46:996-1004.

33. Ikumapayi UN, Boisen N, Hossain MJ, Betts M, Lamin M, Saha D, et al. Identification of Subsets of Enteroaggregative Escherichia coli Associated with Diarrheal Disease among Under 5 Years of Age Children from Rural Gambia. The American journal of tropical medicine and hygiene. 2017;97:997-1004.

34. Isenbarger DW, Hien BT, Ha HT, Ha TT, Bodhidatta L, Pang LW, et al. Prospective study of the incidence of diarrhoea and prevalence of bacterial pathogens in a cohort of Vietnamese children along the Red River. Epidemiology and infection. 2001;127:229-36.

35. Jahan Y, Moriyama M, Hossain S, Rahman M, Ferdous F, Ahmed S, et al. Relation of childhood diarrheal morbidity with the type of tube well used and associated factors of Shigella sonnei diarrhea in rural Bangladesh site of the Global Enteric Multicenter Study. Tropical medicine and health. 2019;47:1-10.

36. Jain P, Chowdhury G, Samajpati S, Basak S, Ganai A, Samanta S, et al. Characterization of non-typhoidal Salmonella isolates from children with acute gastroenteritis, Kolkata, India, during 2000–2016. Brazilian Journal of Microbiology. 2020;51:613-27.

37. Kabayiza J, Andersson M, Nilsson S, Baribwira C, Muhirwa G, Bergström T, et al. Diarrhoeagenic microbes by real-time PCR in Rwandan children under 5 years of age with acute gastroenteritis. Clinical microbiology and infection : the official publication of the European Society of Clinical Microbiology and Infectious Diseases. 2014;20:O1128-35.

38. Kasumba IN, Pulford CV, Perez-Sepulveda BM, Sen S, Sayed N, Permala-Booth J, et al. Characteristics of Salmonella recovered from stools of children enrolled in the Global Enteric Multicenter Study. Clinical infectious diseases : an official publication of the Infectious Diseases Society of America. 2021;73:631-41.

39. Lengerh A, Moges F, Unakal C, Anagaw B. Prevalence, associated risk factors and antimicrobial susceptibility pattern of Campylobacter species among under five diarrheic children at Gondar University Hospital, Northwest Ethiopia. BMC pediatrics. 2013;13:82-.

40. Leting SK, Musyoki SK, Maiyoh GK. Characterization and drug susceptibility pattern of Salmonella and Shigella in children below five years: a cross-sectional study conducted in Lodwar, Turkana County, in Northern Kenya. The Pan African medical journal. 2022;42:13-undefined.

41. Mamuye Y, Metaferia G, Birhanu A, Desta K, Fantaw S. Isolation and Antibiotic Susceptibility Patterns of Shigella and Salmonella among Under 5 Children with Acute Diarrhoea: A Cross-Sectional Study at Selected Public Health Facilities in Addis Ababa, Ethiopia. Clin Microbiol 4: 186. doi: 10.4172/2327-5073.1000186 Page 2 of 7 Clin Microbiol ISSN: 2327-5073 CMO, an open access journal Volume 4• Issue 1• 1000186. level of education A total of. 2015;253:3.

42. Mandomando I, Macete E, Ruiz J, Sanz S, Abacassamo F, Vallès X, et al. Etiology of diarrhea in children younger than 5 years of age admitted in a rural hospital of southern Mozambique. The American journal of tropical medicine and hygiene. 2007;76:522-7.

43. Mason J, Iturriza-Gomara M, O'Brien SJ, Ngwira B, Dove W, Maiden MCJ, et al. Campylobacter Infection in Children in Malawi Is Common and Is Frequently Associated with Enteric Virus Co-Infections. PloS one. 2013;8:e59663-undefined.

44. Mero S, Timonen S, Lääveri T, Løfberg S, Kirveskari J, Ursing J, et al. Prevalence of diarrhoeal pathogens among children under five years of age with and without diarrhoea in Guinea-Bissau. PLoS neglected tropical diseases. 2021;15:e0009709-undefined.

45. Mohakud N, Patra S, Kumar S, Sahu P, Misra N, Shrivastava A. Detection and molecular typing of campylobacter isolates from human and animal faeces in coastal belt of Odisha, India. Indian Journal of Medical Microbiology. 2019;37(3):345-50.

46. Moyo SJ, Gro N, Matee M, Kitundu J, Myrmel H, Mylvaganam H, et al. Age specific aetiological agents of diarrhoea in hospitalized children aged less than five years in Dar es Salaam, Tanzania. BMC pediatrics. 2011;11:19-.

47. Moyo SJ, Maselle SY, Matee M, Langeland N, Mylvaganam H. Identification of diarrheagenic Escherichia coli isolated from infants and children in Dar es Salaam, Tanzania. BMC infectious diseases. 2007;7:92-.

48. Mulatu G, Beyene G, Zeynudin A. Prevalence of Shigella, Salmonella and Cmpylobacter species and their susceptibility patters among under five children with diarrhea in Hawassa town, South Ethiopia. Ethiopian journal of health sciences. 2014;24(2):101.

49. Mwape K, Bosomprah S, Chibesa K, Silwamba S, Luchen CC, Sukwa N, et al. Prevalence of Diarrhoeagenic Escherichia coli among Children Aged between 0–36 Months in Peri-Urban Areas of Lusaka. Microorganisms. 2023;11(11):2790.

50. Nguyen TV, Le Van P, Le Huy C, Gia KN, Weintraub A. Etiology and epidemiology of diarrhea in children in Hanoi, Vietnam. International journal of infectious diseases : IJID : official publication of the International Society for Infectious Diseases. 2006;10:298-308.

51. Nhampossa T, Mandomando I, Acácio S, Quintó L, Vubil D, Ruiz J, et al. Diarrheal Disease in Rural Mozambique: Burden, Risk Factors and Etiology of Diarrheal Disease among Children Aged 0–59 Months Seeking Care at Health Facilities. PloS one. 2015;10:e0119824-undefined.

52. Nigusu Y, Abdissa A, Tesfaw G. Campylobacter Gastroenteritis Among Under-Five Children in Southwest Ethiopia. Infection and drug resistance. 2022;15:2969-79.

53. Nitiema LW, Nordgren J, Ouermi D, Dianou D, Traore AS, Svensson L, et al. Burden of rotavirus and other enteropathogens among children with diarrhea in Burkina Faso. International journal of infectious diseases : IJID : official publication of the International Society for Infectious Diseases. 2011;15:e646-52.

54. Qadri F, Das SK, Faruque ASG, Fuchs GJ, Albert MJ, Sack RB, et al. Prevalence of Toxin Types and Colonization Factors in Enterotoxigenic Escherichia coli Isolated during a 2-Year Period from Diarrheal Patients in Bangladesh. Journal of clinical microbiology. 2000;38:27-31.

55. Randremanana R, Randrianirina F, Gousseff M, Dubois N, Razafindratsimandresy R, Hariniana ER, et al. Case-control study of the etiology of infant diarrheal disease in 14 districts in Madagascar. PloS one. 2012;7:e44533-undefined.

56. Rivera FP, Ochoa TJ, Maves RC, Bernal M, Medina AM, Meza R, et al. Genotypic and Phenotypic Characterization of Enterotoxigenic Escherichia coli Strains Isolated from Peruvian Children. Journal of clinical microbiology. 2010;48:3198-203.

57. Rogawski McQuade ET, Shaheen F, Kabir F, Rizvi A, Platts-Mills JA, Aziz F, et al. Epidemiology of Shigella infections and diarrhea in the first two years of life using culture-independent diagnostics in 8 low-resource settings. PLoS neglected tropical diseases. 2020;14(8):e0008536.

58. Saeed A, Abd H, Sandström G. Microbial aetiology of acute diarrhoea in children under five years of age in Khartoum, Sudan. Journal of medical microbiology. 2015;64:432-7.

59. Sambe-Ba B, Espié E, Faye ME, Timbiné LG, Sembène M, Gassama-Sow A. Community-acquired diarrhea among children and adults in urban settings in Senegal: clinical, epidemiological and microbiological aspects. BMC infectious diseases. 2013;13:580-.

60. Shah MM, Kathiiko C, Wada A, Odoyo E, Bundi M, Miringu G, et al. Prevalence, seasonal variation, and antibiotic resistance pattern of enteric bacterial pathogens among hospitalized diarrheic children in suburban regions of central Kenya. Tropical medicine and health. 2016;44:39-.

61. Shah MM, Odoyo E, Wandera EA, Kathiiko C, Bundi M, Miringu G, et al. Burden of Rotavirus and Enteric Bacterial Pathogens among Children under 5 Years of Age Hospitalized with Diarrhea in Suburban and Rural Areas in Kenya. Japanese journal of infectious diseases. 2017;70:442-7.

62. Shrestha SK, Shrestha J, Mason CJ, Sornsakrin S, Dhakhwa JR, Shrestha BR, et al. Etiology of Acute Diarrheal Disease and Antimicrobial Susceptibility Pattern in Children Younger Than 5 Years Old in Nepal. The American journal of tropical medicine and hygiene. 2022;108:174-80.

63. Singh MK, Bisht D, Goel V, Tomar R, Agrawal A. Detection of bacterial and intestinal parasitic infections among children under 5 years of age with acute gastroenteritis in a tertiary care hospital in NCR region. Santosh University Journal of Health Sciences. 2020;4:87-90.

64. Tafa B, Sewunet T, Tassew H, Asrat D. Isolation and Antimicrobial Susceptibility Patterns of Campylobacter Species among Diarrheic Children at Jimma, Ethiopia. International journal of bacteriology. 2014;2014:560617-.

65. Thompson CN, Phan VT, Le TP, Pham TN, Hoang LP, Ha V, et al. Epidemiological features and risk factors of Salmonella gastroenteritis in children resident in Ho Chi Minh City, Vietnam. Epidemiol Infect. 2013;141(8):1604-13.

66. Tosisa W, Mihret A, Ararsa A, Eguale T, Abebe T. Prevalence and antimicrobial susceptibility of Salmonella and Shigella species isolated from diarrheic children in Ambo town. BMC pediatrics. 2020;20:91-.

67. Uddin MS, Rahman MM, Faruk MO, Talukder A, Hoq MI, Das S, et al. Bacterial gastroenteritis in children below five years of age: a cross-sectional study focused on etiology and drug resistance of Escherichia coli O157, Salmonella spp., and Shigella spp. Bulletin of the National Research Centre. 2021;45:1-7.

68. Vargas M, Gascon J, Casals C, Schellenberg D, Urassa H, Kahigwa E, et al. Etiology of diarrhea in children less than five years of age in Ifakara, Tanzania. The American journal of tropical medicine and hygiene. 2004;70:536-9.

69. Vubil D, Acacio S, Quinto L, Balleste-Delpierre C, Nhampossa T, Kotloff K, et al. Clinical features, risk factors, and impact of antibiotic treatment of diarrhea caused by Shigella in children less than 5 years in Manhica District, rural Mozambique. Infection and Drug Resistance. 2018;11:2095-106.

70. Worku M, Tessema B, Ferede G, Ochieng L, Leliso SA, Mutua F, et al. Campylobacter jejuni and Campylobacter coli infection, determinants and antimicrobial resistance patterns among under-five children with diarrhea in Amhara National Regional State, Northwest Ethiopia. Plos one. 2024;19(7):e0304409.

71. Zachariah OH, Lizzy M, Rose K, Angela MM. Multiple drug resistance of Campylobacter jejuni and Shigella isolated from diarrhoeic children at Kapsabet County referral hospital, Kenya. BMC infectious diseases. 2021;21:109.

Table S 6. Pooled proportion of DEC pathotypes in childhood diarrhea

Subtype	k (studies)	Pooled proportion (%)	95% CI (%)	Prediction interval (%)	I^2
**EAEC**	25	15.8	9.7 – 24.8	1.1 – 76.3	100
**ETEC**	30	8.1	5.0 – 12.9	0.5 – 60.1	100
**EPEC**	32	7.8	4.8 – 12.5	0.4 – 61.8	100
**EIEC**	11	0.5	0.2 – 1.2	0.0 – 6.1	90
**STEC**	11	0.7	0.2 – 2.0	0.0 – 18.5	90

EAEC: Enteroaggregative *E. coli*; ETEC: Enterotoxigenic *E. coli*; EPEC: Enteropathogenic *E. coli*; EIEC: Enteroinvasive *E. coli*; STEC: Shiga toxin-producing *E. coli*

Table S 7. Country specific pooled proportion of enteric pathogens

Country	*Campylobacter* spp. (PP%, 95% CI%, PI%, n)	DEC (PP%, 95% CI%, PI%, n)	*Salmonella* spp. (PP%, 95% CI%, PI%, n)	*Shigella* spp. (PP%, 95% CI%, PI%, n)
**Bangladesh**	17.7 (8.2–34.1), PI: 15.8–19.8, n=2	16.1 (1.7–68.2), PI: 0.7–83.7, n=4	6.1 (1.0–29.0), PI: 0.5–43.7, n=4	21.4 (4.7–60.2), PI: 2.6–73.5, n=4
**Burkina Faso**	1.8 (0.7–4.2), PI: –, n=1	15.5 (0.2–95.7), PI: 5.1–38.8, n=2	4.6 (0.0–96.4), PI: 1.0–18.8, n=2	4.4 (0.3–38.2), PI: 3.0–6.5, n=2
**CAR**	1.5 (0.6–3.6), PI: –, n=1	11.1 (8.2–15.0), PI: –, n=1	3.9 (2.3–6.6), PI: –, n=1	18.3 (14.5–22.8), PI: –, n=1
**Ethiopia**	10.3 (7.4–14.3), PI: 5.3–19.3, n=7	18.6 (6.6–42.4), PI: 7.7–38.6, n=3	5.1 (3.2–8.0), PI: 1.5–16.3, n=11	6.5 (4.6–9.1), PI: 2.3–17.2, n=13
**Gambia**	39.4 (37.1–41.8), PI: –, n=1	51.2 (17.4–83.9), PI: 41.7–60.6, n=2	2.5 (0.0–75.3), PI: 0.8–8.0, n=2	46.2 (43.9–48.6), PI: –, n=1
**Ghana**	–	–	7.5 (4.8–11.6), PI: –, n=1	6.3 (3.8–10.1), PI: –, n=1
**Guinea-Bissau**	51.8 (45.3–58.2), PI: –, n=1	61.4 (54.9–67.5), PI: –, n=1	2.2 (0.9–5.2), PI: –, n=1	27.2 (21.8–33.3), PI: –, n=1
**Haiti**	–	80.2 (71.0–87.0), PI: –, n=1	–	–
**India**	13.3 (0.9–72.1), PI: 7.1–23.3, n=2	6.0 (0.0–100.0), PI: 0.0–97.5, n=2	0.8 (0.3–2.3), PI: 0.2–2.8, n=4	18.7 (2.3–69.6), PI: 2.8–64.5, n=3
**Kenya**	18.0 (9.6–31.3), PI: 10.7–28.7, n=3	25.8 (8.1–57.7), PI: 5.0–69.5, n=4	2.3 (1.2–4.4), PI: 0.7–7.6, n=6	6.2 (1.6–20.9), PI: 0.4–51.1, n=6
**Madagascar**	9.5 (8.4–10.8), PI: –, n=1	13.6 (11.8–15.6), PI: –, n=1	1.5 (1.0–2.1), PI: –, n=1	1.7 (1.3–2.4), PI: –, n=1
**Malawi**	21.4 (19.6–23.3), PI: –, n=1	–	–	–
**Mali**	32.9 (30.6–35.2), PI: –, n=1	47.0 (44.5–49.4), PI: –, n=1	0.4 (0.0–100.0), PI: 0.0–10.6, n=2	27.4 (25.2–29.6), PI: –, n=1
**Morocco**	4.1 (1.7–9.5), PI: –, n=1	58.2 (49.3–66.6), PI: –, n=1	4.1 (1.7–9.5), PI: –, n=1	6.6 (3.3–12.6), PI: –, n=1
**Mozambique**	3.3 (2.2–5.0), PI: 2.2–4.9, n=4	15.8 (3.4–49.7), PI: 1.1–76.4, n=5	1.1 (0.6–1.9), PI: 0.5–2.3, n=5	2.8 (0.4–18.2), PI: 0.2–29.9, n=4
**Nepal**	8.1 (6.7–9.8), PI: –, n=1	7.9 (0.0–100.0), PI: 0.4–65.9, n=2	2.7 (0.4–15.5), PI: 2.1–3.6, n=2	9.9 (0.9–56.9), PI: 1.2–50.7, n=3
**Nigeria**	–	88.3 (81.3–93.0), PI: –, n=1	2.5 (0.8–7.5), PI: –, n=1	–
**Pakistan**	–	–	3.1 (2.3–4.2), PI: –, n=1	18.5 (13.6–24.5), PI: –, n=1
**Peru**	10.1 (0.0–100.0), PI: 0.0–96.9, n=2	5.4 (4.3–7.0), PI: –, n=1	–	4.3 (0.0–98.1), PI: 0.0–96.1, n=3
**Rwanda**	16.7 (14.4–19.3), PI: –, n=1	89.7 (87.5–91.5), PI: –, n=1	6.6 (5.1–8.4), PI: –, n=1	17.5 (15.1–20.2), PI: –, n=1
**Senegal**	0.9 (0.1–6.1), PI: –, n=1	13.4 (8.2–21.0), PI: –, n=1	5.4 (2.4–11.4), PI: –, n=1	7.1 (3.6–13.6), PI: –, n=1
**Sudan**	1.8 (0.9–3.6), PI: –, n=1	7.4 (0.0–100.0), PI: 0.0–97.0, n=2	1.8 (0.0–98.4), PI: 0.2–11.7, n=2	8.6 (2.0–30.3), PI: 6.9–10.6, n=2
**Tanzania**	6.8 (0.9–38.4), PI: 0.4–56.3, n=4	27.5 (6.2–68.5), PI: 1.9–88.2, n=5	1.6 (0.0–40.3), PI: 0.9–2.9, n=2	16.2 (5.2–40.6), PI: 2.3–61.6, n=5
**Vietnam**	6.9 (5.9–8.0), PI: –, n=1	15.9 (3.0–53.2), PI: 3.6–48.9, n=3	5.4 (4.4–6.7), PI: –, n=1	6.1 (4.4–8.2), PI: 5.3–7.0, n=3
**Zambia**	1.1 (0.4–3.4), PI: –, n=1	32.5 (0.0–100.0), PI: 0.3–98.7, n=2	7.7 (5.1–11.6), PI: –, n=1	10.0 (0.0–99.6), PI: 1.4–45.6, n=2

*PP = pooled proportion; CI = 95% confidence interval; PI = 95% prediction interval; n = number of studies. PIs were not estimable for single-study countries (n = 1). For very sparse strata (n ≤ 2), PIs should be interpreted with caution due to instability.

Table S 8. Year specific pooled proportion of enteric pathogens

Year	*Campylobacter* spp. (PP, 95% CI, PI, n)	DEC (PP, 95% CI, PI, n)	*Salmonella* spp. (PP, 95% CI, PI, n)	*Shigella* spp. (PP, 95% CI, PI, n)
**2000**	–	18.0% (16.6–19.5), PI: –, n=1	–	–
**2001**	6.9% (5.9–8.0), PI: –, n=1	6.5% (5.5–7.6), PI: –, n=1	–	6.2% (5.2–7.3), PI: –, n=1
**2004**	2.0% (1.0–3.8), PI: –, n=1	35.7% (31.4–40.2), PI: –, n=1	1.1% (0.5–2.6), PI: –, n=1	21.5% (18.0–25.5), PI: –, n=1
**2006**	–	22.5% (19.3–26.0), PI: –, n=1	–	4.8% (3.3–6.8), PI: –, n=1
**2007**	1.7% (0.9–3.2), PI: –, n=1	22.7% (9.2–46.1), PI: 20.0–25.8, n=2	2.5% (1.4–4.2), PI: –, n=1	0.2% (0.0–1.3), PI: –, n=1
**2008**	–	25.7% (20.7–31.5), PI: –, n=1	–	8.4% (5.6–12.6), PI: –, n=1
**2009**	16.4% (15.0–17.8), PI: –, n=1	–	4.3% (3.6–5.2), PI: –, n=1	4.5% (3.8–5.4), PI: –, n=1
**2010**	1.1% (0.6–1.9), PI: –, n=1	5.4% (4.3–7.0), PI: –, n=1	–	0.1% (0.0–0.6), PI: –, n=1
**2011**	–	15.2% (0.2–94.6), PI: 5.2–36.9, n=2	2.4% (0.1–43.1), PI: 1.4–4.0, n=2	7.2% (0.8–41.5), PI: 1.1–34.6, n=3
**2012**	9.5% (8.4–10.8), PI: –, n=1	5.8% (0.0–99.7), PI: 0.6–37.9, n=2	2.5% (0.5–10.8), PI: 0.7–9.0, n=3	2.7% (0.0–71.3), PI: 0.9–8.0, n=2
**2013**	5.5% (0.5–38.5), PI: 0.3–56.9, n=4	19.0% (1.0–84.4), PI: 10.3–32.4, n=2	7.6% (0.7–47.9), PI: 5.4–10.7, n=2	6.1% (0.4–48.5), PI: 4.1–8.9, n=2
**2014**	11.7% (6.8–19.4), PI: 5.1–24.8, n=5	77.9% (0.1–100), PI: 28.6–96.9, n=2	5.9% (4.2–8.3), PI: 4.8–7.3, n=4	7.3% (2.3–20.9), PI: 1.7–26.8, n=4
**2015**	3.0% (0.1–59.3), PI: 1.3–6.9, n=2	40.4% (15.6–71.2), PI: 17.5–68.3, n=3	3.4% (1.0–10.5), PI: 0.7–14.5, n=4	8.6% (6.0–12.3), PI: 5.1–14.3, n=5
**2016**	1.5% (0.6–3.6), PI: –, n=1	18.7% (0.1–98.0), PI: 5.3–48.6, n=2	2.0% (0.0–85.7), PI: 0.5–7.8, n=2	5.2% (0.0–100), PI: 0.2–61.3, n=2
**2017**	4.9% (0.1–80.4), PI: 0.1–71.2, n=3	26.9% (4.1–76.3), PI: 0.9–93.8, n=5	4.6% (0.8–22.3), PI: 1.0–18.7, n=3	6.1% (1.4–22.2), PI: 0.5–44.2, n=5
**2018**	16.5% (0.0–100), PI: 0.3–93.3, n=2	5.6% (0.1–75.4), PI: 0.2–66.9, n=3	2.4% (0.4–12.1), PI: 0.1–29.8, n=5	7.5% (4.4–12.5), PI: 2.8–18.7, n=6
**2019**	10.8% (1.7–46.2), PI: 2.0–41.4, n=3	33.6% (7.4–76.2), PI: 23.9–44.9, n=2	2.6% (0.3–20.5), PI: 0.4–16.6, n=3	10.5% (0.4–75.6), PI: 0.6–69.4, n=3
**2020**	10.9% (1.8–45.7), PI: 8.4–14.2, n=2	8.0% (0.7–50.9), PI: 0.1–85.5, n=5	2.4% (0.4–14.3), PI: 0.1–38.4, n=5	20.7% (11.7–34.1), PI: 3.8–63.7, n=10
**2021**	28.8% (0.0–100), PI: 3.7–81.1, n=2	13.2% (0.0–100), PI: 0.1–97.8, n=2	1.3% (0.5–3.6), PI: 0.1–15.3, n=9	14.0% (1.6–61.3), PI: 2.1–55.4, n=3
**2022**	8.0% (5.6–11.4), PI: 6.8–9.4, n=3	23.6% (21.3–26.1), PI: –, n=1	2.8% (0.4–18.5), PI: 2.0–3.8, n=2	6.4% (1.7–21.6), PI: 5.3–7.9, n=2
**2023**	23.9% (9.0–50.2), PI: 6.1–60.5, n=4	57.5% (28.7–82.0), PI: 8.1–95.4, n=7	3.2% (1.7–5.9), PI: 1.0–9.7, n=6	18.7% (7.7–38.7), PI: 3.0–63.2, n=6
**2024**	7.0% (4.9–9.8), PI: –, n=1	–	–	–

*PP = pooled proportion; CI = 95% confidence interval; PI = 95% prediction interval; n = number of studies. PIs were not estimable for single-study years (n = 1). For very sparse strata (n ≤ 2), PIs should be interpreted with caution due to instability.

Table S 9. Meta-regression of *Campylobacter* spp., DEC, *Salmonella* spp., and *Shigella* spp. among diarrhea affected children in LMIC's

Variable	Category	Metaregression			
		Univariable		Multivariable	
		Coefficient	p-value	Coefficient	p-value
***Campylobacter* spp.**					
**Continent**			0.66*		
	Africa (Ref)				
	America	0.31	0.73	-	-
	Asia	0.49	0.39	-	-
**Study design**			0.23*		
	Case-control study (Ref)				
	Cohort study	-1.10	0.24	-	-
	Cross-sectional study	-0.63	0.13	-	-
**Source population**			0.24*		
	Community (Ref)				
	Hospital	-0.54	0.24	-	-
**Major diagnostic methods**			<0.01*		
	Culture based methods (Ref)				
	PCR	1.48	<0.01	1.32	<0.01
**Duration of study**			0.30*		
	P50 (4 – 15.5 months) (Ref)				
	P100 (15.6 – 132 months)	0.42	0.30	-	-
**Sample size**			0.70*		
	P50 (41 – 370) (Ref)				
	P100 (371 – 2550)	-0.15	0.70	-	-
**Year**	-	0.09	0.01	0.06	0.03
**DEC**					
**Continent**			0.20*		
	Africa (Ref)				
	America	0.24	0.84	-	-
	Asia	-0.98	0.08	-	-
**Study design**			0.36*		
	Case-control study (Ref)				
	Cohort study	-0.88	0.46	-	-
	Cross-sectional study	-0.68	0.17	-	-
			0.59*		

**Source population**	Community (Ref)				
	Hospital	-0.30	0.59	-	-
**Major diagnostic methods**			0.33*		
	Culture-based methods (Ref)				
	PCR	0.48	0.33	-	-
**Duration of study**			0.60*		
	P50 (3 – 16 months) (Ref)				
	P100 (22 – 60 months)	0.25	0.60	-	-
**Sample size**			0.38*		
	P50 (41 – 437) (Ref)				
	P100 (451 – 2612)	0.42	0.38	-	-
**Year**	-	0.04	0.25	-	-
***Salmonella* spp.**					
**Continent**			0.88*		
	Africa (Ref)				
	Asia	-0.05	0.88		
**Study design**			<0.01*		
	Case-control study (Ref)				
	Cohort study	3.24	<0.01	2.42	<0.01
	Cross-sectional study	0.44	0.09	-0.06	0.83
**Source**			0.83*		
	Community (Ref)				
	Hospital	-0.09	0.83	-	-
**Major diagnostic methods**			0.85*		
	Culture-based methods (Ref)				
	PCR	0.07	0.85	-	-
**Duration of study**			<0.01*		
	P50 (3 – 20 months) (Ref)				
	P100 (22 – 204 months)	-0.85	<0.01	-0.39	0.21
**Sample size**			<0.01*		
	P50 (41 – 437) (Ref)				
	P100 (451 – 9957)	-0.93	<0.01	-0.63	0.04
**Year**	-	-0.02	0.44	-	-
***Shigella* spp.**					
**Continent**			0.20*		
	Africa (Ref)				
	America	-0.31	0.74	-	-

	Asia	0.61	0.09	-	-
**Study design**			0.04*		
	Case-control study (Ref)				
	Cohort study	0.42	0.40	-0.22	0.62
	Cross-sectional study	-0.58	0.09	-0.34	0.22
**Source**			0.04*		
	Community (Ref)				
	Hospital	-0.69	0.04	0.44	0.24
**Major diagnostic methods**			<0.01*		
	Culture-based methods (Ref)				
	PCR	1.62	<0.01	1.61	<0.01
**Duration of study**			0.12*		
	P50 (3 – 12 months) (Ref)				
	P100 (15 – 60 months)	0.47	0.12	-	-
**Sample size**			0.23*		
	P50 (37 – 305) (Ref)				
	P100 (309 – 2550)	-0.37	0.23	-	-
**Year**	-	0.10	<0.01	0.06	0.01

*p-value associated with moderators in the meta-regression model

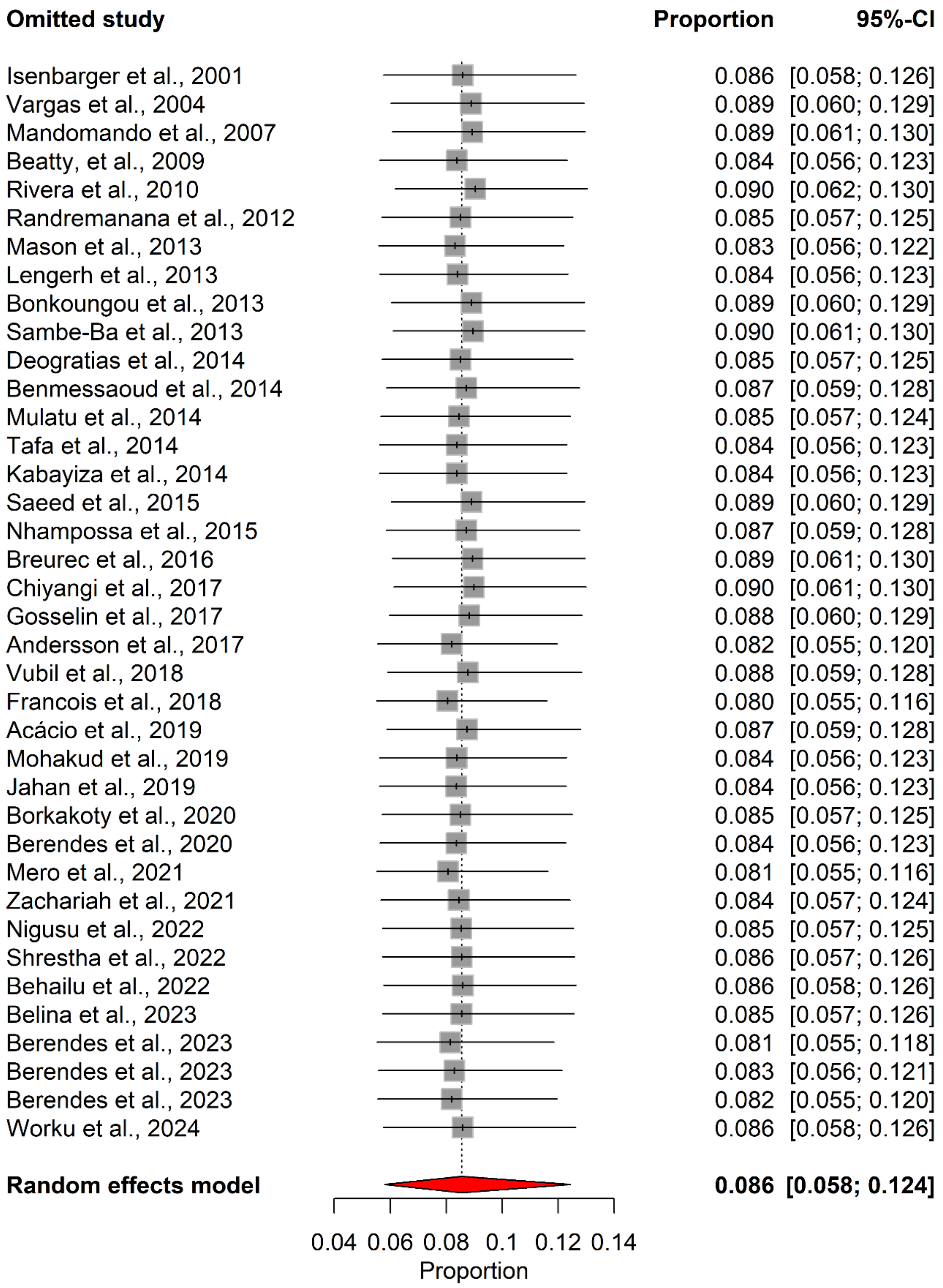

Figure S 2. Leave-one-out (LOO) sensitivity analysis for *Campylobacter* spp.

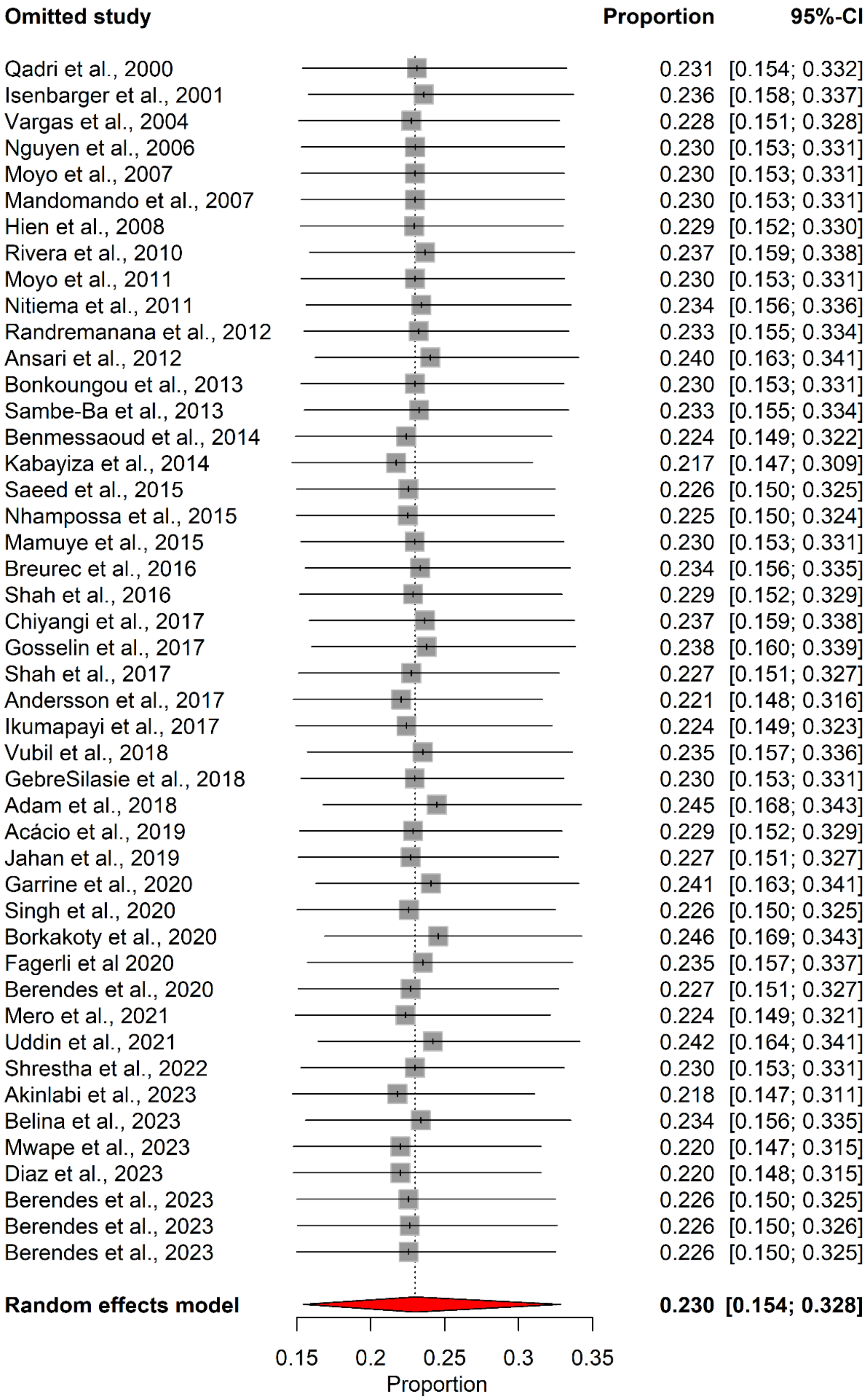

Figure S 3. Leave-one-out (LOO) sensitivity analysis for DEC.

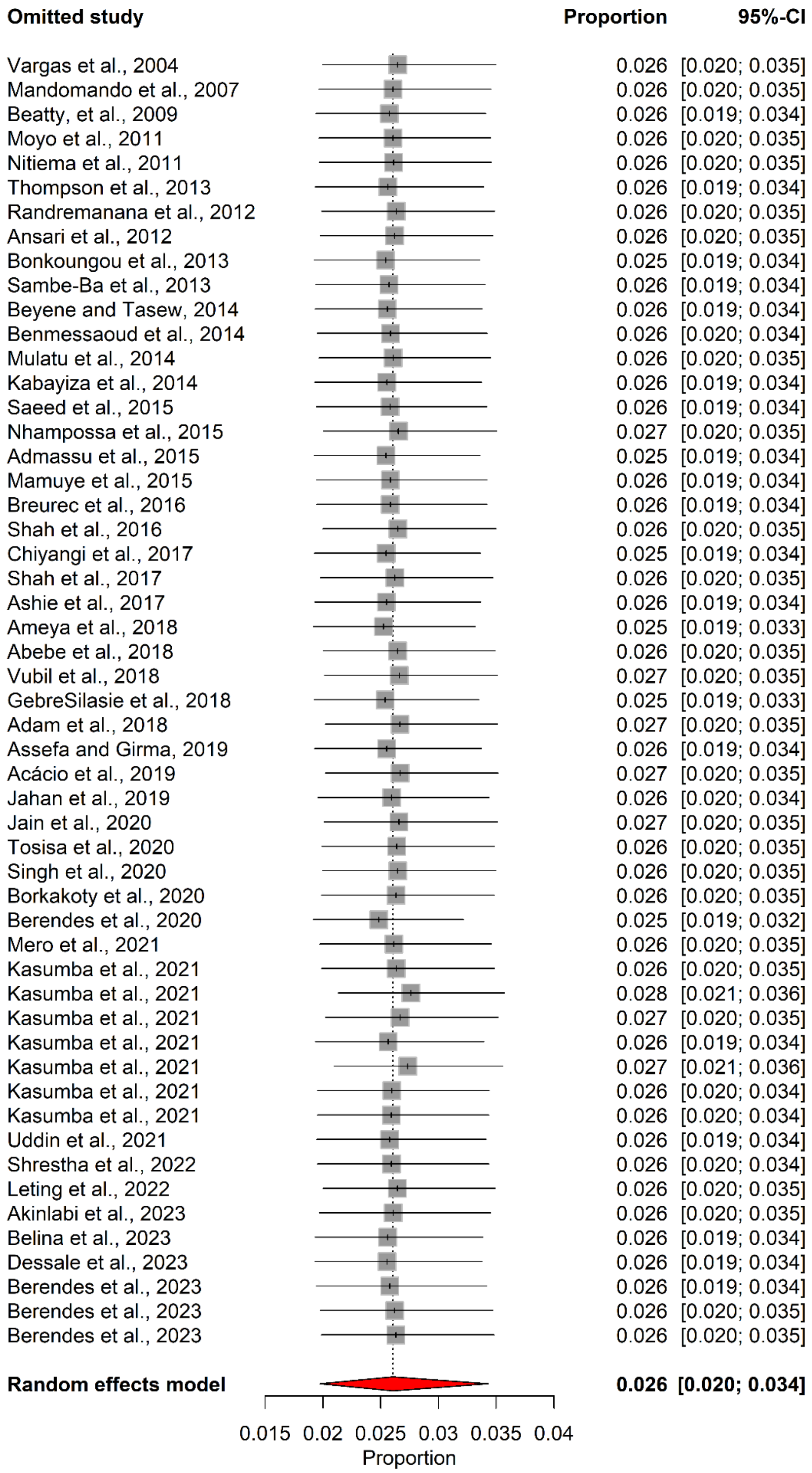

Figure S 4. Leave-one-out (LOO) sensitivity analysis for *Salmonella* spp.

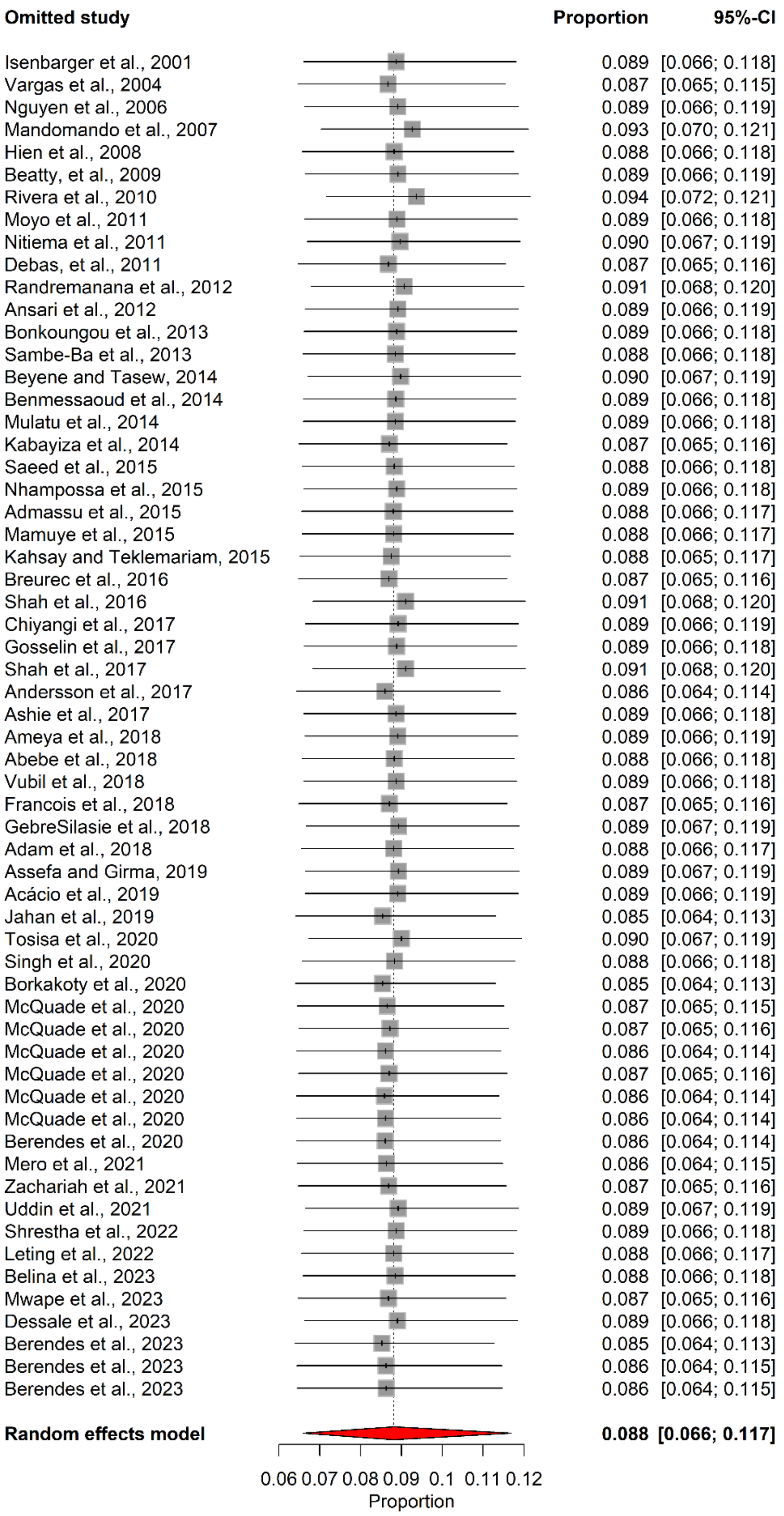

Figure S 5. Leave-one-out (LOO) sensitivity analysis for *Shigella* spp.

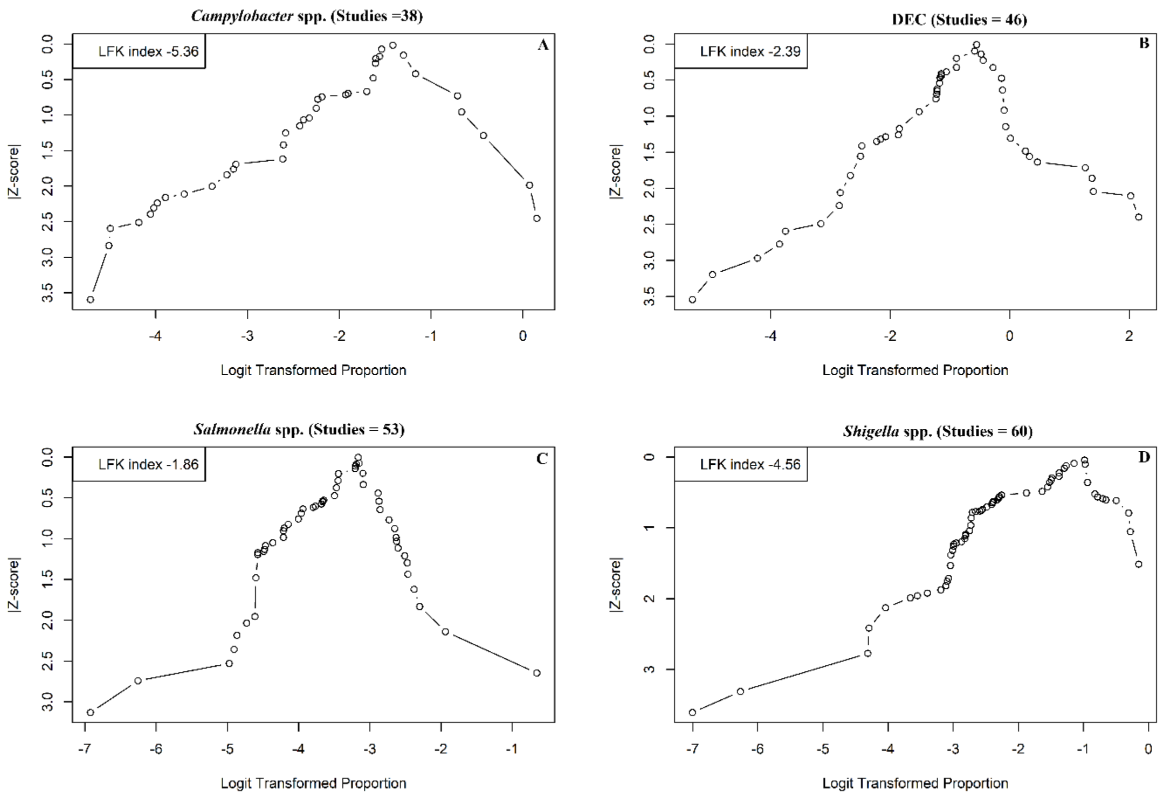

Figure S 6. Doi plot of studies (n=84) included in the meta-analysis, with z-score on the y-axes and proportion for each pathogen on the x-axes: A. *Campylobacter* spp.; B. DEC; C. *Salmonella* spp.; D. *Shigella* spp.

Table S 10. Results of Egger's regression test, rank correlation test, and DOI plot (LFK index) for assessing potential publication bias and small-study effects in the meta-analysis of enteropathogenic bacteria.

Organism	No. of studies (k)	Egger's test (p-value)	Rank correlation (p-value)	LFK index	Interpretation
***Campylobacter* spp.**	38	<0.01	0.05	–5.3	Egger indicates significant asymmetry; Rank borderline; LFK suggests major asymmetry (likely due to heterogeneity)
**DEC**	46	<0.01	0.07	–2.3	Egger indicates significant asymmetry; Rank non-significant; LFK suggests major asymmetry (likely due to heterogeneity)
***Salmonella* spp.**	53	<0.01	0.01	–1.8	Egger and Rank indicate significant asymmetry; LFK suggests minor/moderate asymmetry (likely due to heterogeneity and small-study effects)
***Shigella* spp.**	60	<0.01	0.17	–4.5	Egger indicates significant asymmetry; Rank non-significant; LFK suggests major asymmetry (likely due to heterogeneity and small-study effects)

Note: Egger's regression test is highly sensitive to heterogeneity. Significant results may therefore reflect variance inflation rather than true publication bias, particularly in datasets with high between-study heterogeneity. LFK indices and rank correlation tests were considered alongside Egger's test to provide a balanced assessment.
